# Multi-omics reveals the mechanism of rumen microbiome and its metabolome together with host metabolome participating in the regulation of milk production traits in dairy buffaloes

**DOI:** 10.3389/fmicb.2024.1301292

**Published:** 2024-03-08

**Authors:** Bingxing Jiang, Chaobin Qin, Yixue Xu, Xinhui Song, Yiheng Fu, Ruijia Li, Qingyou Liu, Deshun Shi

**Affiliations:** ^1^School of Animal Science and Technology, Guangxi University, Nanning, China; ^2^School of Life Science and Engineering, Foshan University, Foshan, China

**Keywords:** dairy buffaloes, milk yield, milk fat yield, rumen metagenome, rumen metabolome, serum metabolome

## Abstract

Recently, it has been discovered that certain dairy buffaloes can produce higher milk yield and milk fat yield under the same feeding management conditions, which is a potential new trait. It is unknown to what extent, the rumen microbiome and its metabolites, as well as the host metabolism, contribute to milk yield and milk fat yield. Therefore, we will analyze the rumen microbiome and host-level potential regulatory mechanisms on milk yield and milk fat yield through rumen metagenomics, rumen metabolomics, and serum metabolomics experiments. Microbial metagenomics analysis revealed a significantly higher abundance of several species in the rumen of high-yield dairy buffaloes, which mainly belonged to genera, such as *Prevotella*, *Butyrivibrio*, *Barnesiella*, *Lachnospiraceae*, *Ruminococcus*, and *Bacteroides*. These species contribute to the degradation of diets and improve functions related to fatty acid biosynthesis and lipid metabolism. Furthermore, the rumen of high-yield dairy buffaloes exhibited a lower abundance of methanogenic bacteria and functions, which may produce less methane. Rumen metabolome analysis showed that high-yield dairy buffaloes had significantly higher concentrations of metabolites, including lipids, carbohydrates, and organic acids, as well as volatile fatty acids (VFAs), such as acetic acid and butyric acid. Meanwhile, several *Prevotella*, *Butyrivibrio*, *Barnesiella*, and *Bacteroides* species were significantly positively correlated with these metabolites. Serum metabolome analysis showed that high-yield dairy buffaloes had significantly higher concentrations of metabolites, mainly lipids and organic acids. Meanwhile, several *Prevotella*, *Bacteroides*, *Barnesiella*, *Ruminococcus*, and *Butyrivibrio* species were significantly positively correlated with these metabolites. The combined analysis showed that several species were present, including *Prevotella.*sp.*CAG1031*, *Prevotella.*sp.*HUN102*, *Prevotella.*sp.*KHD1*, *Prevotella.phocaeensis*, *Butyrivibrio.*sp.*AE3009*, *Barnesiella.*sp.*An22*, *Bacteroides.*sp.*CAG927*, and *Bacteroidales.bacterium.52–46*, which may play a crucial role in rumen and host lipid metabolism, contributing to milk yield and milk fat yield. The “omics-explainability” analysis revealed that the rumen microbial composition, functions, metabolites, and serum metabolites contributed 34.04, 47.13, 39.09, and 50.14%, respectively, to milk yield and milk fat yield. These findings demonstrate how the rumen microbiota and host jointly affect milk production traits in dairy buffaloes. This information is essential for developing targeted feeding management strategies to improve the quality and yield of buffalo milk.

## Introduction

As the world’s population continues to grow, meeting the demand for animal products has become a primary concern for national food security (Kearney, 2010). In recent years, the nutritional value of buffalo milk has gained attention; its key nutrients are higher than those of cow and human milk, making buffalo milk an excellent choice for those seeking high-quality milk (Liang et al., 2020). Meanwhile, good quality fat provides the body with energy, including some essential fatty acids that the body cannot synthesize, and is essential for maintaining health and bodily functions, making the quality of fat an important measure of the quality of dairy products (Huang et al., 2019). However, the low milk yield of buffaloes is the main problem, limiting the development of the buffalo milk industry. There are many factors that affect milk quality and yield in dairy buffaloes, including genetic factors (Gernand and Konig, 2014), rumen environment (Wang et al., 2021), feeding management (Thompson et al., 2022), and feed digestion (Leduc et al., 2021). It has been widely reported that milk yield is usually negatively correlated with milk fat yield (Lai and Liu, 2019), but recently we have found that under the same feeding management conditions, some dairy buffaloes can exhibit both relatively high milk yield and milk fat yield compared with others, which would be a potential new trait for buffalo dairy producers to select (Xue et al., 2019).

The rumen is a digestive chamber found in ruminants that houses a diverse community of microorganisms, including bacteria, archaea, eukaryotes, protozoa, and viruses (Zhao et al., 2023). First, rumen microbes are influenced by various host factors, including diet, age, breed, and genetics. Meanwhile, ruminal microbes have a symbiotic relationship with their hosts and are able to obtain nutrients from crude fiber, which is indigestible to humans. They can break down cellulose and pentosan in feed into usable organic acids, mainly acetic, propionic, and butyric acids (often referred to as volatile fatty acids or VFAs). These VFAs are absorbed in large quantities through the rumen wall and can meet 60–80% of the energy needs of ruminants (Huws et al., 2018). Studies have shown that this affects feed efficiency (Auffret et al., 2020) and methane emissions (Gonzalez-Recio et al., 2023) from cattle. Second, the digestion of rumen microorganisms is crucial in ruminant nutrition and closely linked to the breakdown and synthesis of nutrients, such as sugars, proteins, and lipids. Rumen microorganisms are also able to synthesize nutrients such as essential amino acids, essential fatty acids, and B vitamins for use by the host, all of which determine the quality of animal products (Weimer, 2015). Thus, we hypothesize that the rumen microbiome can directly or indirectly affect milk yield and milk fat yield in dairy buffaloes.

The biosynthesis of milk in dairy buffaloes is a complicated biological process that involves not only the rumen but also host metabolic processes. Microorganisms break down nitrogenous material in the diet into peptides and amino acids, and these compounds are then utilized for milk protein synthesis (Agregan et al., 2021). Additionally, starch and fiber in the diet are broken down by microorganisms into glucose, which is used for the synthesis of lactose (Lin et al., 2016). The fatty acids are used to synthesize milk fat that is present in four main sources: 1. Rumen microorganisms break down lipids from the diet, including triglycerides, glycolipids, and phospholipids. This process mainly produces long-chain fatty acids that are absorbed during the circulation and used directly by the mammary gland of the host. 2. The rumen microorganisms break down and ferment crude fiber in the diet into volatile fatty acids, primarily acetic acid and butyric acid. These acids then enter the mammary gland through the bloodstream, providing energy for the synthesis of fatty acids (Liu et al., 2021). 3. There is also a small proportion of fatty acids in the blood derived from fat mobilization by the host organism, and the fatty acids obtained from the breakdown of body fat are also absorbed and utilized by the host mammary gland (Loften et al., 2014). 4. Ruminal microorganisms can synthesize microbial lipids, including specific branched chain and polyunsaturated fatty acids, from volatile fatty acids. These fatty acids are digested in the small intestine after the microorganisms die and are then absorbed into the host’s circulation for direct use (Shah et al., 2022). Moreover, for the final formation of the entire dairy product, the rumen microorganisms and host play a vital role. Recent studies have shown that milk production traits in dairy cows can be influenced by rumen microbes and their metabolites, as well as host metabolism, and the metabolite group has been found to have a greater degree of influence than the microbial group (Xue et al., 2020). However, this relationship has not yet been studied in dairy buffaloes. Therefore, it is speculated that rumen microbes may initially affect the host’s metabolism by influencing diet digestion and the metabolic environment within the rumen. Subsequently, this may further affect the milk yield and milk fat yield. In our study, we divided dairy buffaloes into two groups, the high-yield (dairy buffaloes with high milk yield and milk fat yield) group of dairy buffaloes and the low-yield (dairy buffaloes with low milk yield and milk fat yield) group of dairy buffaloes, which were studied mainly for rumen metagenomics, rumen metabolomics, and serum metabolomics, testing to address the following questions: Do the rumen microbiome (composition and functions), microbial metabolites, and the host metabolites contribute to milk yield and milk fat yield? If so, do they affect this trait equally? The rumen microbiome and metabolome, as well as the host metabolome, were compared between dairy buffaloes with high-yield and low-yield, and the contributions of the above three omics layers to milk yield and milk fat yield were calculated. The research above will not only serve as a reference for early selection of high-quality dairy buffaloes but will also aid in improving the rumen environment through genetic selection and feeding management that will result in the production of high-quality dairy buffalo milk.

## Results

### Characterization of phenotypes

In this experiment, from a group of 226 Murrah dairy buffaloes, 12 dairy buffaloes with the highest yield (dairy buffaloes with high milk yield and milk fat yield; HH group) and 12 dairy buffaloes with the lowest yield (dairy buffaloes with low milk yield and milk fat yield; LL group) were selected for analyses of rumen metagenome, rumen metabolome, and serum metabolome. In the phenotypic data, milk yield (*p* < 0.01), milk fat percentage (*p* < 0.01), and milk fat yield (*p* < 0.01) were significantly different between the two groups (Supplementary Table S1).

### The rumen metagenome

**Figure 1:**
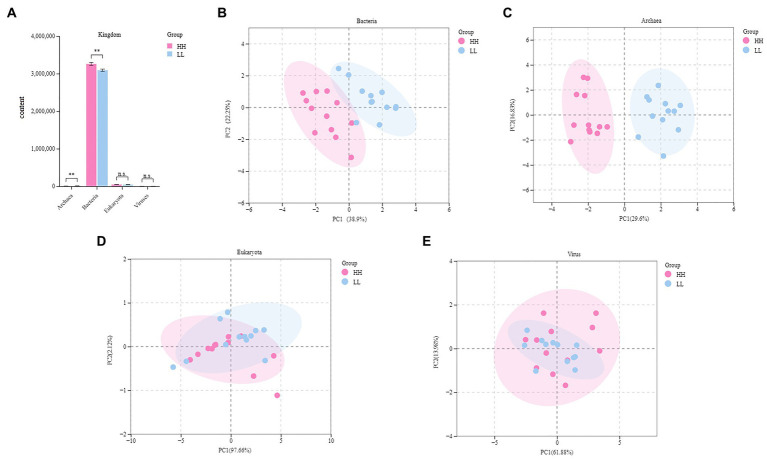
Rumen microbial compositional profiles of HH and LL dairy buffaloes. **(A)** Comparison of microbial domains between HH and LL dairy buffaloes. **(B)** Bacterial compositional profiles of HH and LL rumen samples based on species visualized using principal coordinate analysis (PCoA). **(C)** Archaeal compositional profiles of HH and LL rumen samples based on species visualized using PCoA. **(D)** Eukaryotic compositional profiles of HH and LL rumen samples based on species visualized using PCoA. **(E)** Viral compositional profiles of HH and LL rumen samples based on species visualized using PCoA. *p < 0.05, **p < 0.01.

Metagenome sequencing generated 257137.91 raw data (10714.08 ± 495.24) per sample. After quality control and removal of host genes, 255829.8 data were obtained, with (10673.61 ± 500.73) data per sample. After *de novo* assembly, the total length of Scaftigs was 13,459,416,214 bp, and the total length of N50 was 22,149 bp, with (922.86 ± 39.63) bp per sample. The total length of N90 was 13,247 bp, with (551.96 ± 5.31) bp per sample (Supplementary Table S2). The overall results of the rumen metagenome showed a composition of (82.3 ± 1.7%) bacteria, (1.3 ± 0.4%) eukaryotes, (0.4 ± 0.1%) archaea, (0.1 ± 0.02%) viruses, (15.6 ± 1.3%) unknown material, and (0.2 ± 0.04%) unclassified material (Supplementary Figure S1). Upon comparing the rumen microbial domains of the two groups, it was found that the bacteria and archaea significantly differed (*p* < 0.05). The relative levels of bacteria were higher in the rumen of HH dairy buffaloes, while the relative levels of archaea were higher in the rumen of LL dairy buffaloes (Figure 1A). The PERMANOVA analysis (permutational multivariate analysis of variance) indicated significant differences between bacteria and archaea (*p* < 0.01), while no significant differences were observed between eukaryota and viruses (*p* > 0.05; Supplementary Table S3). Principal coordinate analysis (PCoA) showed separation between the two groups based on bacterial (Figure 1B) and archaeal (Figure 1C) species, while no separation was found based on eukaryotic (Figure 1D) or viral (Figure 1E) species. Therefore, the downstream comparative analysis of the rumen microbiota will concentrate solely on bacteria and archaea.

### Compositional profiles of the rumen microbiome and taxonomic differences between the HH and LL dairy buffaloes

**Figure 2:**
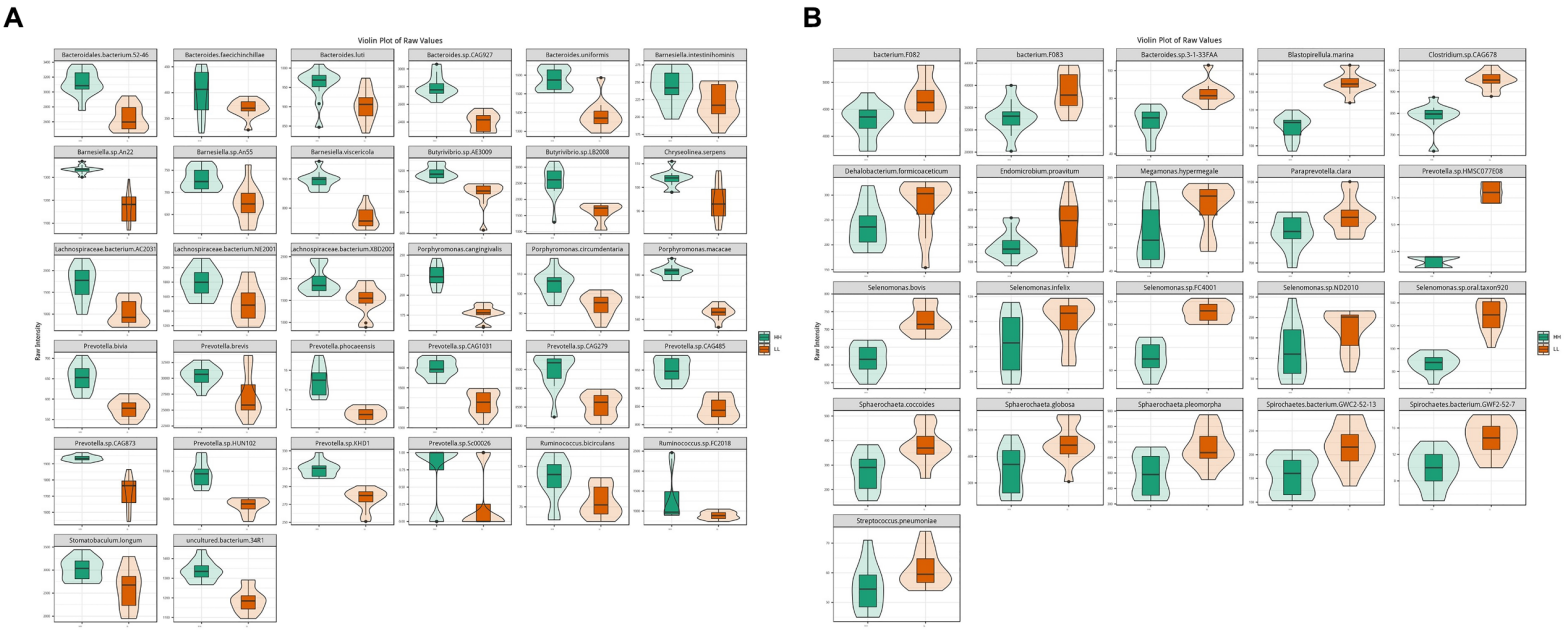
Differential rumen bacterial species between HH and LL dairy buffaloes. **(A)** Significantly different bacterial species in the rumen of HH dairy buffaloes. **(B)** Significantly different bacterial species in the rumen of LL dairy buffaloes. LDA > 2, *p* < 0.05.

According to metagenome data, the main dominant bacterial phyla between the two groups included *Bacteroidetes* (45.2 ± 3.0%), *Firmicutes* (37 ± 2.8%), and *Proteobacteria* (0.9 ± 0.1%). The main dominant bacterial genera included *Prevotella* (24.3 ± 2.3%), *Bacteroides* (4.6 ± 0.3%), and *Clostridium* (3.1 ± 0.4%). The main dominant bacterial species included *Clostridiales bacterium* (3.5 ± 0.3%), *Prevotella ruminicola* (2.3 ± 0.3%), *Prevotella.*sp.*ne3005* (2.0 ± 0.2%), *Bacteroidales bacterium WCE2004* (1.8 ± 0.1%), *Prevotella.*sp.*tc2-28* (1.5 ± 0.2%), and *Prevotella.*sp.*tf2-5* (1.2 ± 0.1%). Through comparative analysis of differential abundance, it was found that at the phylum level, *Bacteroidetes* and *Proteobacteria* were more abundant in the rumen of HH dairy buffaloes, while *Firmicutes* were more abundant in the rumen of LL dairy buffaloes. At the genus level, *Prevotella* was more abundant in the rumen of HH dairy buffaloes, while *Clostridium* and *Bacteroides* were more abundant in the rumen of LL dairy buffaloes (Supplementary Figure S2). At the species level, 32 microorganisms were found to be more abundant in the rumen of HH dairy buffaloes, including 10 species of *Prevotella*, 5 species of *Bacteroides*, 4 species of *Barnesiella*, 3 species of *Lachnospiraceae*, 3 species of *Porphyromonas*, 2 species of *Ruminococcus*, 2 species of *Butyrivibrio*, 1 species of *Chryseolinea*, 1 species of *Stomatobaculum*, and 1 species of uncultured bacterium (Figure 2A). Additionally, 21 microorganisms were found to be more abundant in the rumen of LL dairy buffaloes (LDA > 2, *p* < 0.05; Figure 2B).

**Figure 3:**
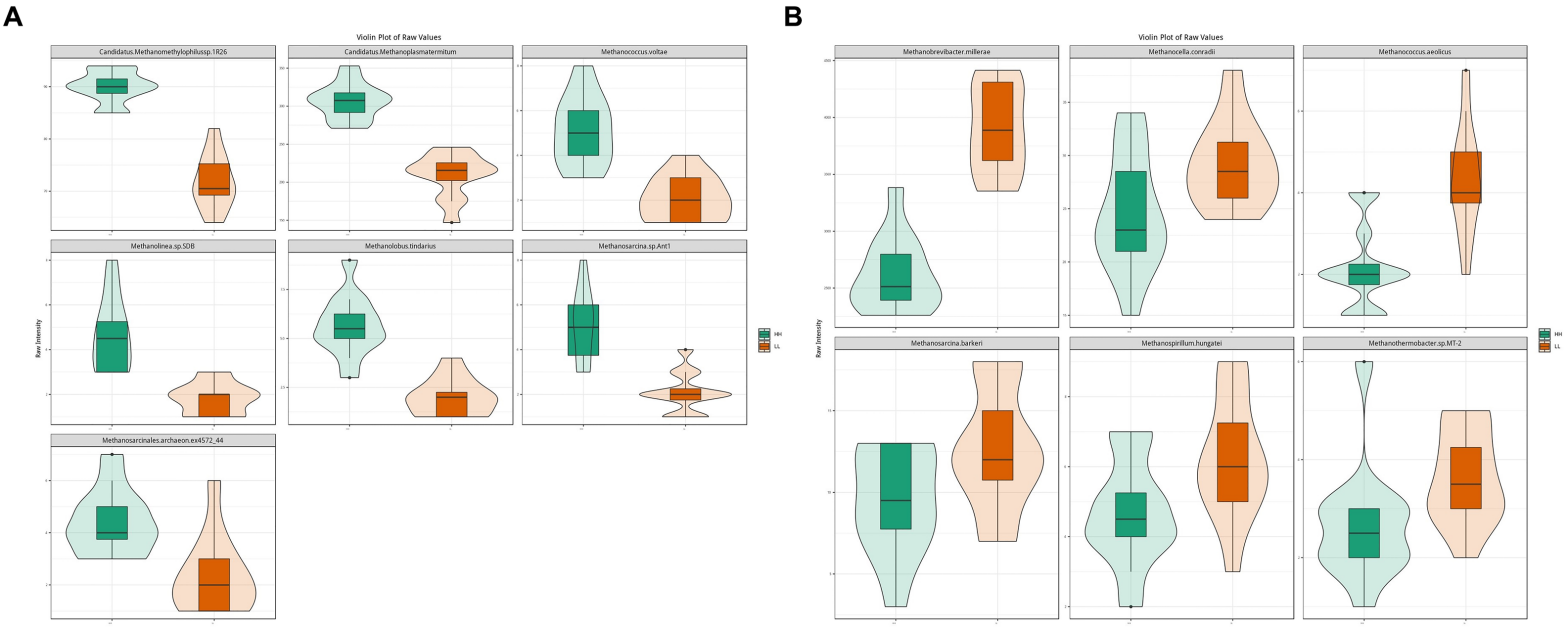
Differential rumen archaeal species between HH and LL dairy buffaloes. **(A)** Significantly different archaeal species in the rumen of HH dairy buffaloes. **(B)** Significantly different archaeal species in the rumen of LL dairy buffaloes. LDA > 2, *p* < 0.05.

The results for archaea showed that at the phylum level, *Euryarchaeota* (96.3 ± 0.7%) was the most abundant archaeal phylum, which was significantly higher in the rumen of LL dairy buffaloes. At the genus level, *Methanobrevibacter* (51 ± 10.8%) was the most abundant archaeal genus, which was significantly higher in the rumen of LL dairy buffaloes, while most of the remaining differential genera were higher in the rumen of HH dairy buffaloes. At the species level, *Methanogenic archaeon mixed culture ISO4-G1* (19.6 ± 6.1%) and *Methanobrevibacter millerae* (14 ± 5.7%) were the most abundant archaea (Supplementary Figure S3). When comparing the differential archaeal species between the two groups, it was found that 7 archaeal species were significantly more abundant in the rumen of HH dairy buffaloes (Figure 3A), while 6 archaeal species were significantly more abundant in the rumen of LL dairy buffaloes (LDA > 2, *p* < 0.05; Figure 3B).

### Functional profiles of the rumen microbiome and differential functions between the HH and LL dairy buffaloes

**Figure 4:**
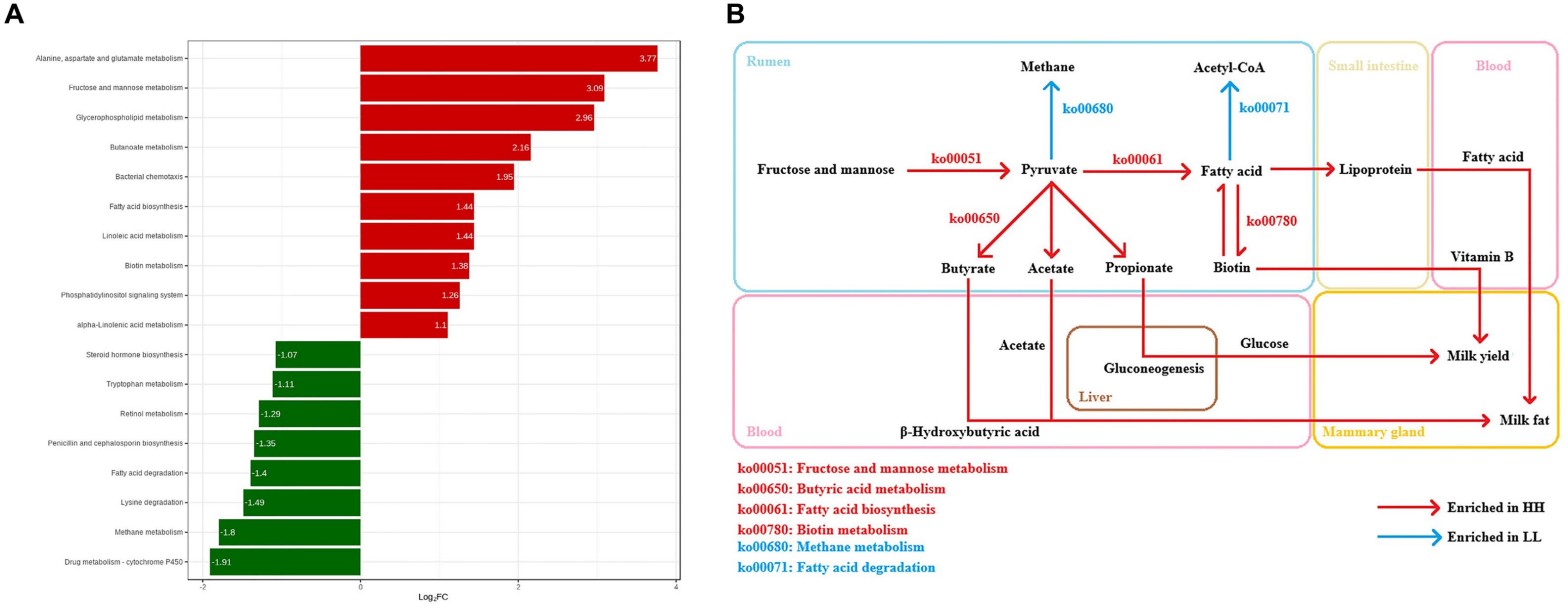
Differential KEGG functions between HH and LL dairy buffaloes. **(A)** HH/LL fold change of significantly enriched metabolic pathways. **(B)** Differential microbial functions involved in carbohydrate metabolism, vitamin metabolism, lipid metabolism, and energy metabolism in the rumen of HH and LL dairy buffaloes. LDA > 2, *p* < 0.05.

In this experiment, the functions of the rumen microbiome were determined by the Kyoto Encyclopedia of Genes and Genomes (KEGG) profiles and genes encoding CAZymes (Supplementary Figure S4). The KEGG functional profile identified 387 metabolic third-level pathways. Of these, 228 are part of the rumen microbial metabolic pathways (Supplementary Table S4). These pathways belonged to four first-level categories, including “Metabolism” (57.3 ± 0.6%), “Cellular processes” (9.5 ± 0.2%), “Environmental information processing” (11.1 ± 0.3%), and “Genetic information processing” (22 ± 0.3%). At the second-level categories, 23 s-level pathways of rumen microbial metabolism were identified, of which “Carbohydrate metabolism” (15.6 ± 0.1%), “Amino acid metabolism” (10.6 ± 0.1%), “Nucleotide metabolism” (8.3 ± 0.1%), “Energy metabolism” (7.0 ± 0.1%), “Translation” (6.7 ± 0.2%), and “Cofactor and vitamin metabolism” (6.5 ± 0.1%) were the highest relative content (Supplementary Figure S5). When comparing the identified KEGG functions, there were 10 third-level pathways and 9 functional modules that were significantly enriched in the rumen microbiome of HH dairy buffaloes (Figure 4A), while 8 third-level pathways and 8 functional modules were significantly enriched in the rumen microbiome of LL dairy buffaloes (LDA > 2, *p* < 0.05; Supplementary Figure S6). Regarding carbohydrate, vitamin, lipid, and energy metabolism, 7 third-level pathways and 7 functional modules were enriched in the rumen microbiome of HH dairy buffaloes. The 7 third-level pathways included “fatty acid biosynthesis” (ko00061), “butyric acid metabolism” (ko00650), “glycerophospholipid metabolism” (ko00564), “fructose and mannose metabolism” (ko00051), “linoleic acid metabolism” (ko00591), “alpha-linolenic acid metabolism” (ko00592), and “biotin metabolism” (ko00780). The 7 functional modules included “fatty acid biosynthesis, initiation” (M00082), “fatty acid biosynthesis, elongation” (M00083), “ketone body biosynthesis” (M00088), “phosphatidylethanolamine (PE) biosynthesis” (M00093), “phosphatidylcholine biosynthesis, PE = > PC” (M00091), “biotin biosynthesis” (M00123), and “pimeloyl ACP biosynthesis, BioC-BioH pathway” (M00572); 4 third-level pathways and 6 functional modules were enriched in the rumen microbiome of LL dairy buffaloes. The four third-level pathways included “steroid hormone biosynthesis” (ko00140), “fatty acid degradation” (ko00071), “methane metabolism” (ko00680), and “retinol metabolism” (ko00830). The six functional modules included “beta oxidation” (M00087), “acetyl coenzyme A pathway” (M00422), “xylulose monophosphate pathway” (M00344), “serine pathway” (M00346), and “methanogenesis” (M00357, M00567) (Figure 4B).

For the CAZymes functional profile, 306 genes encoding CAZymes were identified (Supplementary Table S5), including 7 auxiliary activities (AAs), 70 carbohydrate-binding modules (CBMs), 14 carbohydrate esterases (CEs), 133 glycoside hydrolases (GHs), 62 glycosyltransferases (GTs), and 20 polysaccharide lyases (PLs). Of these, the gene encoding GH2 (7.1 ± 0.1%) was the most dominant, followed by the gene encoding GH3 (6.3 ± 0.1%), the gene encoding GH43 (6.2 ± 0.2%), the gene encoding GT2 (4.3 ± 0.2%), and the gene encoding GH13 (3.1 ± 0.1%). By comparing the data between the two groups, in the second-level categories, 1 AAs, 6 CBMs, 3 CEs, 14 GHs, 9 GTs, and 2 PLs were enriched in the rumen microbiome of HH dairy buffaloes, while 0 AAs, 4 CBMs, 0 CEs, 8 GHs, 7 GTs, and 1 PLs were enriched in the rumen microbiome of LL dairy buffaloes (LDA > 2, *p* < 0.05; Supplementary Figure S7). Glycoside hydrolases (GHs) and carbohydrate-binding modules (CBMs) are among the CAZyme genes primarily involved in the degradation of plant cellulose in diets. They are closely related to cellulases, xylanases, and other enzymes. Glycosyltransferases (GTs) are carbohydrate synthesis enzymes encoded by CAZyme genes. They are primarily associated with rumen fermentation and the production of volatile fatty acids. Higher abundance of GHs, CBMs, and GTs was observed in the rumen microbiome of HH dairy buffaloes.

### Associations between microbial species and microbial functions

**Figure 5:**
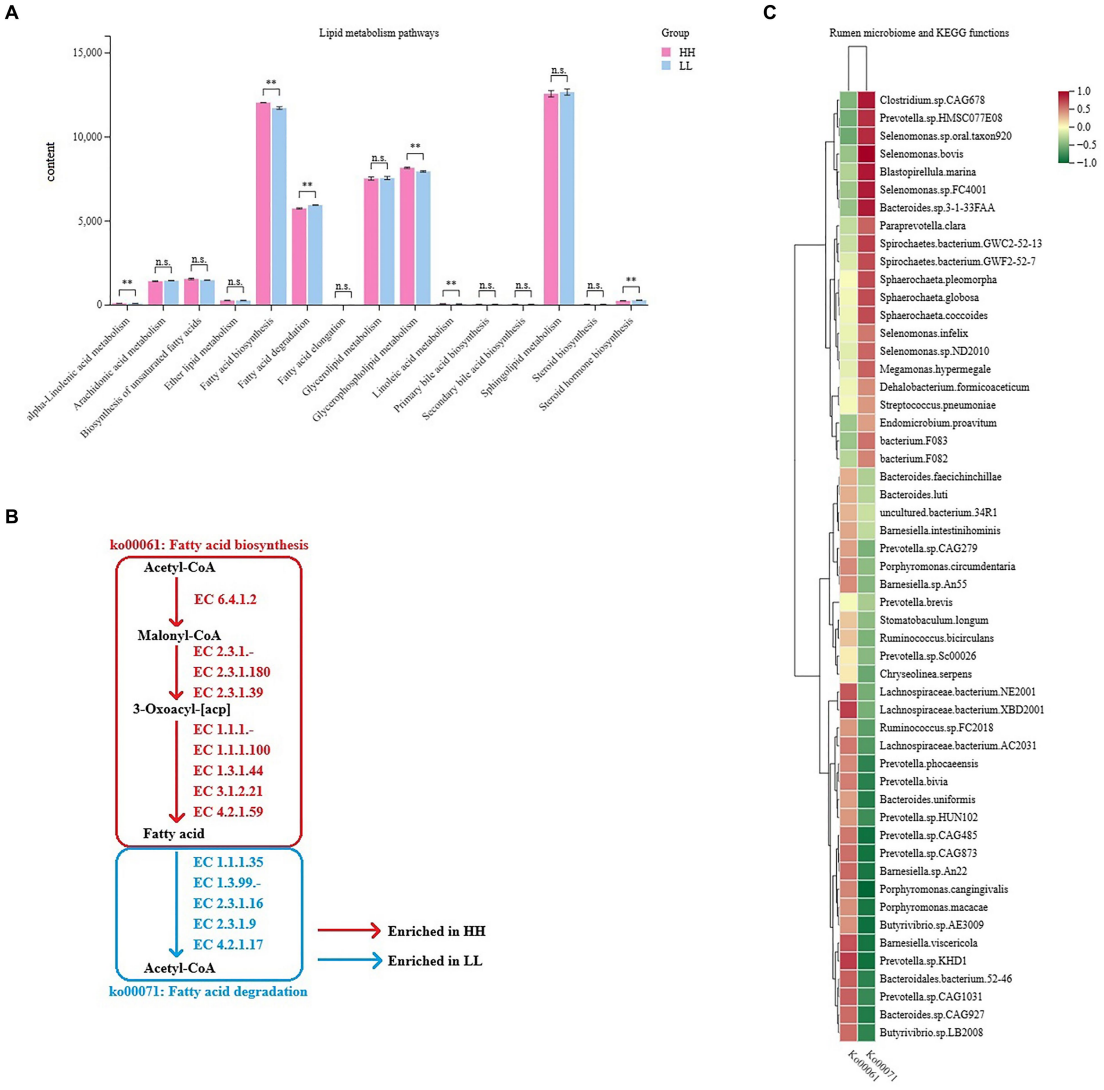
Microbial functions and species involved in lipid metabolism in the rumen of HH and LL dairy buffaloes. **(A)** Comparison of microbial functions involved in lipid metabolism in the rumen of HH and LL dairy buffaloes. **(B)** Fatty acid biosynthesis and degradation pathways. **(C)** Spearman’s correlation between significantly different bacterial species and two fatty acid metabolism pathways. LDA > 2, *p* < 0.05, * *p* < 0.05, ** *p* < 0.01. The color depth (red: positive, green: negative) is proportional to the correlation strength. Spearman’s R > 0.5 or < − 0.5 and *p* < 0.05 are considered as significant.

As milk fat yield is an important indicator of milk production traits, we further focused on the functions of lipid metabolism in the rumen microbiome. The rumen metagenome results showed 15 pathways regarding lipid metabolism between the two groups, 6 of which differed significantly between the 2 groups (LDA  >  2, *p*  <  0.05; Figure 5A), including “fatty acid biosynthesis,” “glycerophospholipid metabolism,” “linoleic acid metabolism,” “alpha-linolenic acid metabolism,” “steroid hormone biosynthesis,” and “fatty acid degradation.” We found two important pathways involved in lipid metabolism, which were “fatty acid biosynthesis” (ko00061, enriched in the rumen microbiome of HH dairy buffaloes) and “fatty acid degradation” (ko00071, enriched in the rumen microbiome of LL dairy buffaloes), and these pathways showed a converse enrichment between the HH and LL groups (Figure 5B). The abundances of genes encoding enzymes involved in these two pathways were also compared, showing that the abundances of genes encoding enzymes involved in fatty acid biosynthesis were all significantly enriched in the rumen microbiome of HH dairy buffaloes, while the abundances of genes encoding enzymes involved in fatty acid degradation were all significantly higher in the rumen microbiome of LL dairy buffaloes (*p* < 0.05; Supplementary Figure S8). Spearman’s rank correlation network between bacterial species and those two fatty acid metabolism pathways was then created to explore how rumen bacterial species could affect the microbial fatty acid functions. In total, 16 species showed a significant positive correlation with the fatty acid biosynthesis pathway and a significant negative correlation with the fatty acid degradation pathway, including *Prevotella.bivia*, *Prevotella.*sp.*CAG485*, *Prevotella.*sp.*CAG873*, *Prevotella.*sp.*KHD1*, *Prevotella.*sp.*CAG1031*, *Prevotella.*sp.*HUN102*, *Prevotella.phocaeensis*, *Lachnospiraceae.bacterium.NE2001*, *Lachnospiraceae.bacterium.XBD2001*, *Lachnospiraceae.bacterium.AC2031*, *Bacteroides.*sp.*CAG927*, *Bacteroidales.bacterium.52–46*, *Barnesiella.*sp.*An22*, *Barnesiella.viscericola*, *Butyrivibrio.*sp.*AE3009*, and *Butyrivibrio.*sp.*LB2008* (*R* > 0.5, *p* < 0.05; Figure 5C).

### Rumen metabolome and serum metabolome

**Figure 6:**
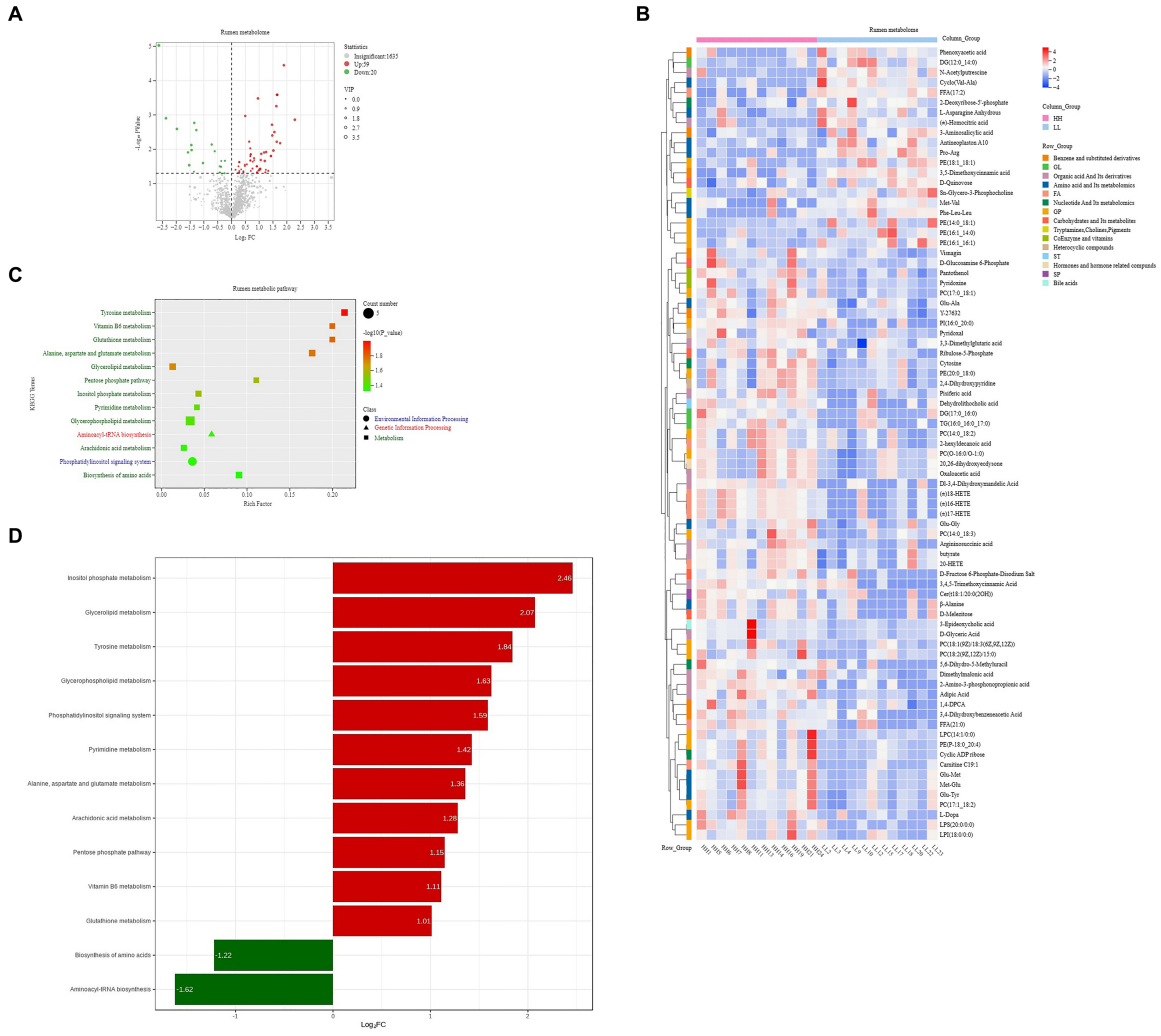
Rumen metabolome of HH and LL dairy buffaloes. **(A)** Comparison of rumen metabolome in HH and LL dairy buffaloes. **(B)** Significantly different rumen metabolites between HH and LL dairy buffaloes. **(C)** Pathway enrichment analysis performed using the significantly different rumen metabolites between HH and LL dairy buffaloes. **(D)** HH/LL fold change of significantly enriched metabolic pathways. The color depth (red: high, blue: low) is proportional to the relative concentrations. *p* < 0.05 was considered as significant. Only significant differences (*p* < 0.05) are displayed.

A total of 1,714 compounds were identified in the rumen metabolome; after t-test and VIP filtering for the relative concentrations of rumen metabolites, 79 metabolites were significantly different between the two groups of dairy buffaloes (Figure 6A), with 59 metabolites significantly higher in the rumen of HH dairy buffaloes and 20 metabolites significantly higher in the rumen of LL dairy buffaloes (VIP > 1, *p* < 0.05; Figure 6B). Based on these 79 significantly different rumen metabolites, analysis of the metabolic pathways revealed enrichment in 13 metabolic pathways (Figure 6C), with “tyrosine metabolism,” “vitamin B6 metabolism,” and “glutathione metabolism” being the significantly different pathways (*p* < 0.01; Figure 6D).

**Figure 7:**
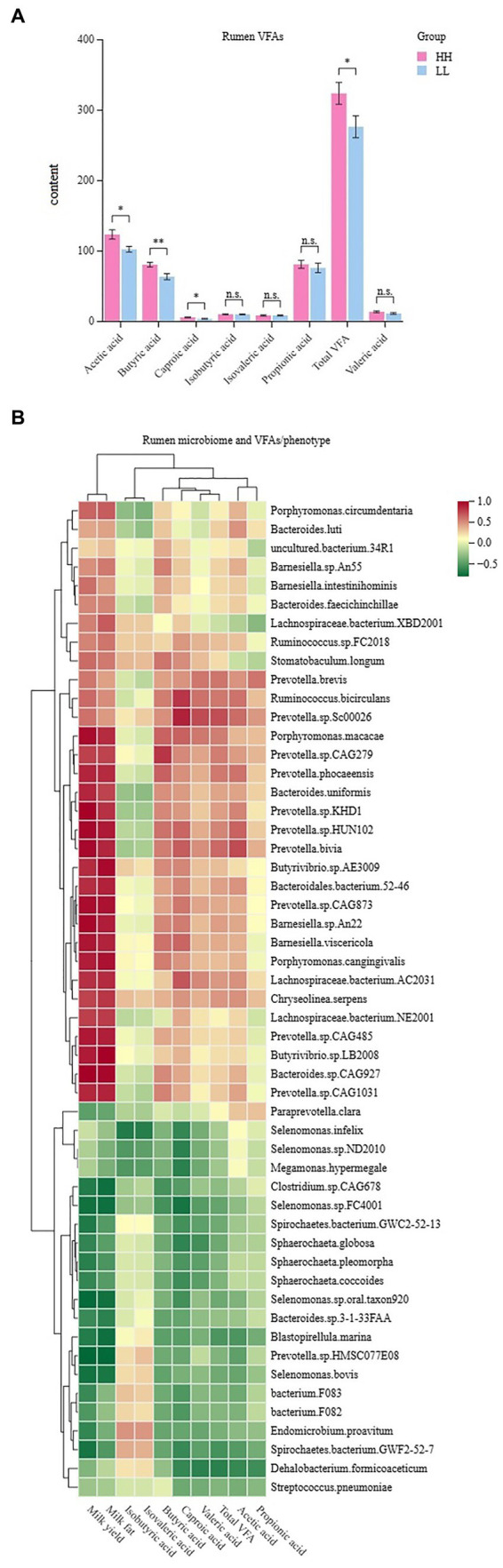
Rumen volatile fatty acids (VFAs) of HH and LL dairy buffaloes. **(A)** Comparison of the concentrations of VFAs in the rumen of HH and LL dairy buffaloes. **(B)** Spearman’s correlation between significantly different bacterial species and VFAs and phenotypes. *p < 0.05, **p < 0.01. The color depth (red: positive, green: negative) is proportional to the correlation strength. Spearman’s R > 0.5 or < − 0.5 and *p* < 0.05 are considered as significant.

The concentrations of volatile fatty acids (VFAs) in the rumen were measured, and seven VFAs were found. The results showed that acetic acid, propionic acid, and butyric acid had the highest concentrations of VFAs in the rumen of dairy buffaloes in both groups. The concentration of total VFAs in the rumen of HH dairy buffaloes was significantly higher than that of LL dairy buffaloes. The concentrations of acetic acid, butyric acid, and capric acid in the rumen of HH dairy buffaloes were significantly higher than those of LL dairy buffaloes (*p* < 0.05), and the concentrations of propionic and valeric acids in the rumen of HH dairy buffaloes were slightly higher than those of LL dairy buffaloes (Figure 7A). Spearman’s correlation results indicate that most microorganisms enriched in the rumen of HH dairy buffaloes were positively correlated with phenotype (milk yield and milk fat yield), acetic acid concentration, butyric acid concentration, and total VFA concentration (*R* > 0.5, *p* < 0.05; Figure 7B).

**Figure 8:**
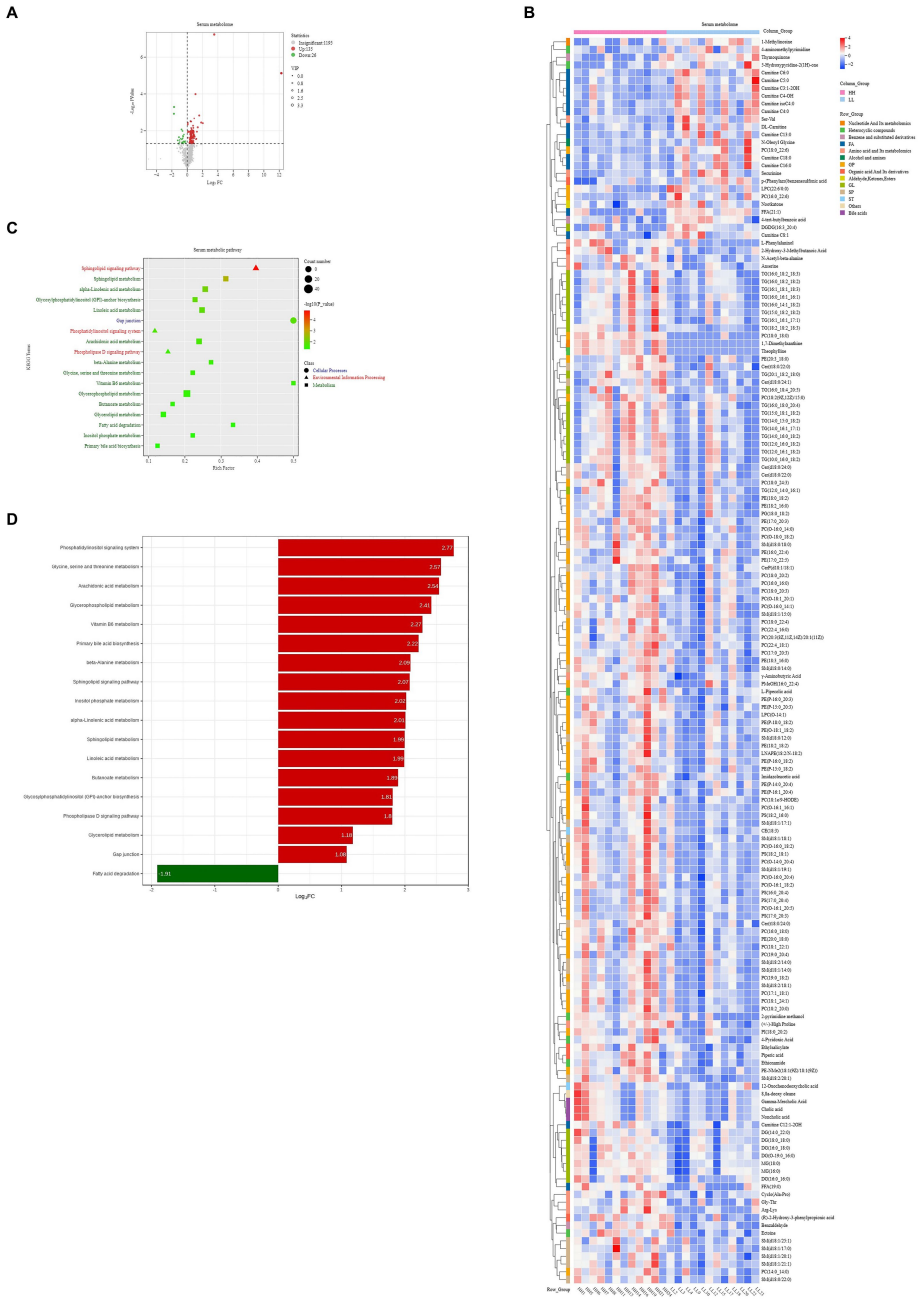
Serum metabolome of HH and LL dairy buffaloes. **(A)** Comparison of serum metabolome in HH and LL dairy buffaloes. **(B)** Significantly different serum metabolites between HH and LL dairy buffaloes. **(C)** Pathway enrichment analysis is performed using the significantly different serum metabolites between HH and LL dairy buffaloes. **(D)** HH/LL fold change of significantly enriched metabolic pathways. The color depth (red: high, blue: low) is proportional to the relative concentrations. *p* < 0.05 was considered as significant. Only significant differences (*p* < 0.05) are displayed.

For the serum metabolome, we identified 1,356 compounds. Comparative analysis shows that 161 metabolites were significantly different between the two groups of dairy buffaloes (Figure 8A), with 135 metabolites significantly higher in the serum of HH dairy buffaloes and 26 metabolites significantly higher in the serum of LL dairy buffaloes (VIP > 1, *p* < 0.05; Figure 8B). Analysis of the metabolic pathways based on these 161 significantly different serum metabolites revealed enrichment in 18 metabolic pathways (Figure 8C), with “sphingolipid metabolism,” “alpha-linolenic acid metabolism,” and “sphingolipid signaling pathway” being the significantly different pathways (*p* < 0.01; Figure 8D).

To identify whether the metabolites in rumen could be related to those in the serum, we compared the rumen and serum metabolome, including the significantly different metabolites and metabolic pathways between the two groups. The Venn diagram of differential metabolites showed that PC (18:2(9Z,12Z)/15:0) and PE(20,0_18,0) were shared by both rumen and serum of HH dairy buffaloes. For the differential metabolic pathways, six pathways were common in both the rumen and serum of HH dairy buffaloes, including “vitamin B6 metabolism,” “phosphatidylinositol signaling system,” “glycerophospholipid metabolism,” “arachidonic acid metabolism,” “glycerolipid metabolism,” and “inositol phosphate metabolism” (Supplementary Figure S9).

### Relationship between metabolites and phenotypes

**Figure 9:**
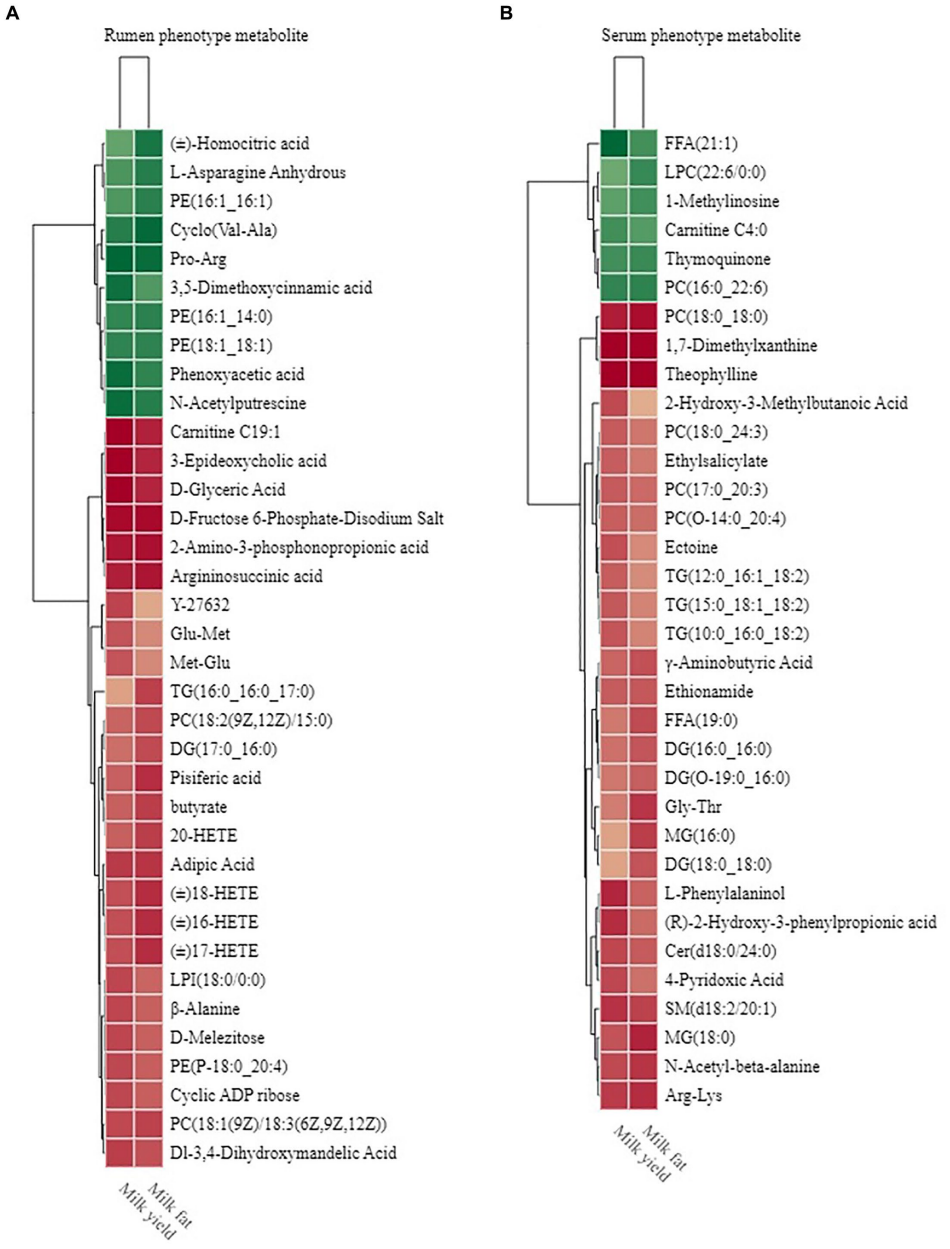
Phenotype-associated metabolites of HH and LL dairy buffaloes. **(A)** Spearman’s correlation between significantly different rumen metabolites and phenotypes. **(B)** Spearman’s correlation between significantly different serum metabolites and phenotypes. The color depth (red: positive, green: negative) is proportional to the correlation strength. Spearman’s R > 0.5 or < − 0.5 and *p* < 0.05 are considered as significant. Only strong (Spearman’s R of > 0.5 or < − 0.5) and significant (*p* < 0.05) correlations are displayed.

The rumen metabolome was also used for phenotype (milk yield and milk fat yield) association analysis; the results of Spearman’s correlation between differential rumen metabolites and phenotype (milk yield and milk fat yield) showed that 26 rumen metabolites were significantly positively correlated with milk yield and milk fat yield, and 10 rumen metabolites were significantly negatively correlated with milk yield and milk fat yield (*R* > 0.5, *p* < 0.05; Figure 9A). These metabolites participate in six metabolic pathways, including “biosynthesis of amino acids,” “alanine, aspartate, and glutamate metabolism,” “arachidonic acid metabolism,” “glycerolipid metabolism,” “glycerophospholipid metabolism,” and “tyrosine metabolism.”

Similarly, the results of Spearman’s correlation between differential host serum metabolites and phenotype (milk yield and milk fat yield) showed that 28 serum metabolites were significantly positively correlated with milk yield and milk fat yield, and 6 serum metabolites were significantly negatively correlated with milk yield and milk fat yield (*R* > 0.5, *p* < 0.05; Figure 9B). These metabolites participate in 9 metabolic pathways, including “glycerophospholipid metabolism,” “arachidonic acid metabolism,” “linoleic acid metabolism,” “alpha-linolenic acid metabolism,” “glycerolipid metabolism,” “sphingolipid metabolism,” “vitamin B6 metabolism,” “sphingolipid signaling pathway,” and “beta-Alanine metabolism.”

We defined these metabolites into phenotype-associated metabolites for further correlation analysis. The Venn diagram of phenotype-associated metabolites showed that “glycerophospholipid metabolism,” “glycerolipid metabolism,” and “arachidonic acid metabolism” were shared by both rumen and serum phenotype-associated metabolites of HH dairy buffaloes (Supplementary Figure S10).

### Relationship between rumen microbiome, rumen metabolome, and serum metabolome and their explainabilities for phenotype

**Figure 10:**
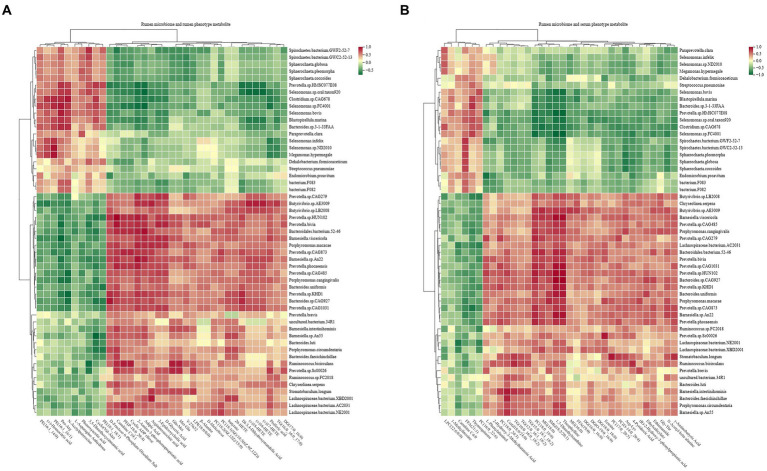
Interactions between rumen metagenome, rumen metabolome, and serum metabolome. **(A)** Spearman’s correlations between rumen microbiota and phenotype-associated metabolites in the rumen. **(B)** Spearman’s correlations between rumen microbiota and phenotype-associated metabolites in serum. The color depth (red: positive, green: negative) is proportional to the correlation strength. Spearman’s R > 0.5 or < − 0.5 and *p* < 0.05 are considered as significant.

Spearman’s rank correlations between the rumen microbiota and rumen phenotype-associated metabolites were assessed, with the results revealing 590 significant correlations (*R* > 0.5, *p* < 0.05; Figure 10A). Among the 590 correlations, significantly positive correlations existed among mainly 11 species (*Prevotella.*sp.*HUN102*, *Prevotella.bivia*, *Prevotella.*sp.*CAG873*, *Prevotella.phocaeensis*, *Prevotella.*sp.*CAG485*, *Prevotella.*sp.*CAG1031*, *Prevotella.*sp.*KHD1*, *Butyrivibrio.*sp.*AE3009*, *Barnesiella.*sp.*An22*, *Bacteroides.*sp.*CAG927*, and *Bacteroidales.bacterium.52–46*), lipids, carbohydrates, and organic acids.

Similarly, the study assessed Spearman’s rank correlations between the rumen microbiota and serum phenotype-associated metabolites, and the results showed 531 significant correlations (*R* > 0.5, *p* < 0.05; Figure 10B). Among the 531 correlations, mainly 10 species (*Prevotella.*sp.*CAG1031*, *Prevotella.*sp.*HUN102*, *Prevotella.*sp.*KHD1*, *Prevotella.phocaeensis*, *Prevotella.*sp.*CAG279*, *Bacteroides.*sp.*CAG927*, *Bacteroidales.bacterium.52–46*, *Barnesiella.*sp.*An22*, *Ruminococcus.bicirculans*, and *Butyrivibrio.*sp.*AE3009*) showed significantly positive correlations with lipids and organic acids.

Significant correlations were also found between the phenotype-associated metabolites and the 17 functional modules of the rumen microbiome (Supplementary Figure S11).

**Figure 11:**
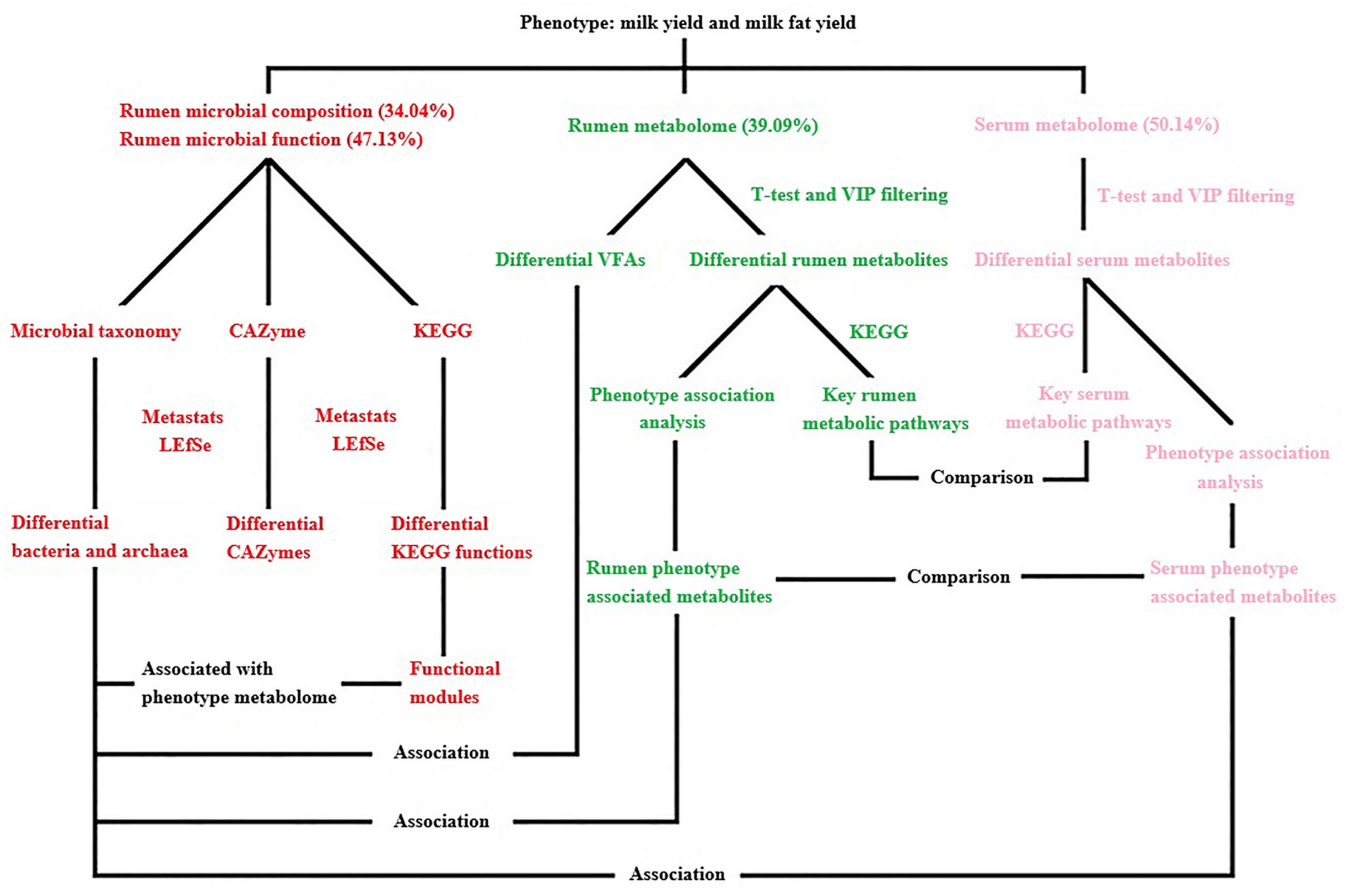
Overview of the workflow. Rumen microbial species (bacteria and archaea) and functions (CAZymes and KEGG functions) were compared between two phenotype (milk yield and milk fat yield) groups (HH and LL). Rumen metabolites were compared between two groups, and the key rumen metabolic pathways were enriched based on the significantly different metabolites, the significantly different rumen metabolites were also separated into two groups that were either positively or negatively correlated with phenotype (milk yield and milk fat yield), defined as the “rumen phenotype-associated metabolites.” Concentrations of volatile fatty acids (VFAs) in the rumen were compared between two groups, and associations between significantly different VFAs and significantly different bacterial species. Serum metabolites were compared between two groups, and the key serum metabolic pathways were enriched based on the significantly different metabolites, the significantly different serum metabolites were also separated into two groups that were either positively or negatively correlated with phenotype, defined as the “serum phenotype-associated metabolites.” The rumen and serum metabolome were compared, including the significantly different metabolites, phenotype-associated metabolites, and differential metabolite-enriched pathways between two groups. The rumen and serum phenotype-associated metabolites were associated with rumen microbiome, including the significantly different rumen microbial species and functional modules between two groups. The proportion of variance in phenotype explained by the rumen microbial composition, rumen microbial functions, rumen metabolome, and serum metabolome (defined as omics-explainability) was estimated.

A linear mixed model was used to calculate the impact of rumen microbial composition, functions, metabolites, and serum metabolites on milk yield and milk fat yield in dairy buffaloes (Supplementary Table S6). The phenotypic variation explained by the rumen microbial composition, rumen microbial functions, rumen metabolome, and serum metabolome was 34.04, 47.13, 39.09, and 50.14%, respectively (Figure 11).

## Discussion

We investigated the mechanisms by which rumen microorganisms, their metabolism, and host metabolism affect milk yield and milk fat yield in dairy buffaloes by integrating the results of multi-omics analysis and estimated the contributions of the rumen microbial composition, functions, metabolites, and serum metabolites to the variations in this trait.

In rumen metagenome experiments, bacteria were the most abundant group in the rumen of dairy buffaloes, which was consistent with previous microbial studies (Peng et al., 2019), and we found that the significantly different microorganisms in the HH and LL dairy buffaloes were mainly bacteria. Bacteria play a significant role in the degradation and fermentation of diets in the rumen of ruminants, providing energy to the animal organism, which suggests that bacteria have a crucial impact on the milk yield and milk fat yield compared with other microbial kingdoms (Bickhart and Weimer, 2018). At the bacterial species level, we identified several significantly more abundant species in the rumen of HH dairy buffaloes, which mainly belonged to genera, such as *Prevotella*, *Bacteroides*, *Butyrivibrio*, *Barnesiella*, *Lachnospiraceae*, and *Ruminococcus*. First, *Prevotella*, one of the core genera, is quite abundant in the rumen of dairy buffaloes. This genus is able to use starch and protein in the diet to produce more succinic and acetic acids (Betancur-Murillo et al., 2022). Second, another significantly higher genus in the rumen of HH dairy buffaloes is *Barnesiella.* This genus is not only the main volatile fatty acid-producing bacteria but is also closely associated with the production of bile acids that facilitate the transport and absorption of fatty acids in the body (Mu et al., 2021). In addition to the aforementioned genera, *Bacteroides*, *Butyrivibrio*, *Lachnospiraceae*, and *Ruminococcus* are also common bacteria that produce volatile fatty acids. These bacteria were found to be positively correlated with volatile fatty acid concentrations and phenotype (milk yield and milk fat yield), indicating their significant role in the biosynthesis of volatile fatty acids and contribution to milk yield and milk fat yield. The Spearman’s correlation results showed that several *Prevotella*, *Barnesiella*, *Bacteroides*, and *Ruminococcus* species showed positive correlations (*R* > 0.5, *p* < 0.05) with the concentrations of acetic acid, butyric acid, and total VFAs, suggesting that may be the key microbial species responsible for the higher concentrations of acetic and butyric acids in the rumen of HH dairy buffaloes. On the other hand, we found that the species with higher relative abundance in the rumen of LL dairy buffaloes mainly belonged to genera, such as *Selenomonas*, *Sphaerochaeta*, and *Spirochaetes*. The genus *Selenomonas* is capable of fermenting glucose to produce acetic acid and propionic acid, but it has also been reported to be the dominant group contributing to methane production (Kornel, 2015). Research has demonstrated a positive correlation between the genus *Sphaerochaeta* and the production of methane and VFA (Zhao et al., 2018). Additionally, the genus *Spirochaetes* has been found to break down propionic acid, butyric acid, and valeric acid into acetic acid for the consumption by methanogenic bacteria (Long et al., 2021). These findings suggest that the rumen of LL dairy buffaloes may expend more energy for methane production, which could contribute to a reduction in milk yield and milk fat yield. Regarding archaea, we found a higher abundance of genus-level *Methanobrevibacter* and species-level *Methanobrevibacter millerae* in the rumen of LL dairy buffaloes, suggesting that LL dairy buffaloes may produce more methane, leading to less-efficient milk production (Shabat et al., 2016). Although only bacteria and archaea show significant differences in overall microbial structure, we cannot ignore the remaining small fraction of eukaryotes, viruses, and protozoa in the rumen environment. For instance, fungi can aid in the breakdown of structurally complex crystalline cellulose by assisting bacteria, and protozoa can modulate the internal rumen environment by phagocytosis bacteria and fungi (Guo et al., 2023). In conclusion, their action on the host, their degradation of diet fiber in the rumen, and their interaction with bacteria are all likely to be important factors influencing milk production traits in dairy buffaloes, which may warrant further studies in the future.

The two groups of dairy buffaloes exhibited differences in microbial functions due to variations in rumen microbial composition. The analysis of KEGG functions shows that the rumen of HH dairy buffaloes is enriched with more metabolic functions regarding carbohydrates, vitamins, lipids, and energy. For instance, the rumen microbiome of HH dairy buffaloes appears to be more proficient in degrading carbohydrates as evidenced by the metabolic pathways “fructose and mannose metabolism” (ko00051), “butyric acid metabolism” (ko00650), and “fatty acid biosynthesis” (ko00061) involving the pyruvate metabolic pathways, resulting in a higher yield of hydrolysis products and pyruvate. Meanwhile, the analysis of CAZyme functions revealed that the abundance of genes encoding plant cellulose-degrading enzymes (GHs and CBMs) in the diet was higher in the rumen of HH dairy buffaloes; the main enrichment was observed in GH13, GH25, GH53, and CBM26, which can express amylase, glucanase, cellulase, xylanase, mannanase, and galactanase indicated that the rumen microbiota of HH dairy buffaloes is more capable of degrading complex substrates in the diet. Furthermore, the higher abundance of genes encoding the primary enzymes involved in carbohydrate synthesis (GTs) and the concentration of the primary VFAs were significantly greater in the rumen of HH dairy buffaloes, suggesting that the microorganisms in the rumen of HH dairy buffaloes were more efficient in producing VFAs through hydrolysis products or pyruvate, resulting in increased energy availability for lactogenesis in dairy buffaloes and improved milk production traits (Xue et al., 2020). In contrast, the rumen microbiome of LL dairy buffalo showed a significant enrichment in the “methane metabolism” (ko00680) pathway, and genes encoding related enzymes involved in methanogenesis, with a higher abundance of EC 2.1.1.86, a methyltransferase that transfers methyl from the coenzyme tetrahydromethanopterin to coenzyme M (CoM), which is required for methane production. Similarly, EC 2.8.4.1 is more abundant, which is a methyl coenzyme M reductase responsible for the final step of methanogenesis in methanogenic metabolism (Chen et al., 2020). These results suggest that in the rumen of LL dairy buffaloes, the energy produced by microbial degradation and fermentation of the diet is utilized more by methane metabolic processes, resulting in lower energy from VFAs (Shi et al., 2014). Both the concentration of VFAs and methane emission in the rumen of ruminants have been reported to be closely related to feed conversion efficiency (Myer et al., 2015; Li and Guan, 2017), so we speculated that to some extent, the rumen microbiome affects feed efficiency, and HH dairy buffaloes may have higher feed conversion efficiency than LL dairy buffaloes. Future measurements of methane emissions and feed conversion efficiency are needed to validate this hypothesis.

Regarding lipid metabolism functions, “linoleic acid metabolism” (ko00591), “alpha-linolenic acid metabolism” (ko00592), and “glycerophospholipid metabolism” (ko00564) are significantly enriched in the rumen microbiome of HH dairy buffaloes and genes encoding related enzymes involved in lipolysis, with a higher abundance of EC 3.1.1.4 and EC 3.1.4.4, a class of phospholipases with hydrolytic effects on phospholipids and triglyceride structures (Lv et al., 2022), suggesting that the rumen microbiome of HH dairy buffaloes may have a greater ability to break down fat in the diet and facilitate lipid transport, resulting in more precursors and energy being available. In addition, rumen microbes synthesize up to 20% of the host animal’s fat requirements, and the enrichment of the “fatty acid biosynthesis” (ko00061) functions in the rumen of HH dairy buffaloes suggests that more microbial lipids may be synthesized in their rumen, and these lipids are then digested in the small intestine and provide the host with a fatty acid pool (Martinez-Alvaro et al., 2022), which, in turn, provides more precursors for milk fat synthesis in the mammary gland. On the other hand, the function “fatty acid degradation” (ko00071), which is enriched in the rumen of LL dairy buffaloes, not only affects the amount of fatty acids reaching the small intestine but has also been shown to cause reduced growth performance in pigs (Meyer et al., 2020). Spearman’s correlation results show that most of the species positively correlated with the metabolic pathways of “fatty acid biosynthesis” and negatively correlated with the pathway of “fatty acid degradation,” which belong to genera, such as *Prevotella*, *Lachnospiraceae*, *Bacteroides*, *Butyrivibrio*, and *Barnesiella*, suggesting that these species may play an important role in the biosynthesis of fatty acids, which is the first report in a study of the rumen microbiome of dairy buffaloes. In the future, research should be conducted using *in vitro* simulated rumen environment cultures to detect the functions of active microorganisms and intracellular energy changes, and this will help determine the specific roles of these species in lipid metabolism processes.

Our study has identified another important metabolic pathway which is vitamin metabolism. Ruminant rumen microbes synthesize vitamin B and vitamin K, which can function as precursors to coenzyme factors involved in basic metabolic processes, such as fatty acid synthesis, amino acid synthesis, and gluconeogenesis to meet their growth and development needs (Jiang et al., 2022). “Biotin metabolism” (ko00780) is a metabolic pathway of vitamin metabolism that is significantly enriched in the rumen microbiome of HH dairy buffaloes; biotin mainly involved as a cofactor in the transfer of CO_2_ in carboxylases, such as acetyl CoA carboxylase can catalyze the synthesis of malonic acid monoacyl CoA from acetyl CoA and CO_2_, providing a two-carbon compound for fatty acid synthesis (Chao et al., 2022). Numerous studies have reported that supplementing additional B vitamins in the diet can significantly enhance milk yield in cows, including milk component yield, suggesting that excellent milk production traits also depend on higher levels of vitamin B (Du et al., 2021). Therefore, it is possible that the rumen microbes of HH dairy buffaloes are able to produce more vitamin B, which may be one of the reasons for their higher milk and milk fat yield. Furthermore, we have identified a metabolic pathway that is significantly enriched in the rumen microbiome of high-producing dairy buffaloes: “Bacterial chemotaxis” (ko02030) for cellular processes. Bacterial chemotaxis is primarily interpreted as a foraging strategy, whereby bacteria adjust their movements toward more growth-friendly nutrients, which can also contribute to the expansion of microenvironmental colonization and the maintenance of bacterial diversity (Colin et al., 2021). Although the exact ecological role is unknown, we hypothesize that rumen microbes of HH dairy buffaloes may be better at sensing and finding favorable nutrients. In the future, further testing of vitamin B production and microbial flagellar motility proteins is necessary to provide a better understanding of the contribution of these functions to milk yield and milk fat yield.

As the outcome of microbiome compositional and functional differences, differences in rumen metabolites and serum metabolites between the two groups were found in this study. In the rumen metabolome, the metabolites that were in higher concentrations in the rumen of HH dairy buffaloes compared with the LL dairy buffaloes included mainly lipids, organic acids, coenzymes vitamins, and carbohydrates. First, the rumen of HH dairy buffaloes showed higher concentrations of D-glyceric acid, D-glucosamine 6-phosphate, and ribulose-5-phosphate, and these metabolites are mainly involved in the “pentose phosphate pathway” (ko00030) and “glutathione metabolism” (ko00480), suggesting that more energy may be produced in the rumen and made available to the host animal (Allen and Piantoni, 2014). Second, the rumen of HH dairy buffaloes contained higher concentrations of diglycerides, triglycerides, phosphatidylinositol, phosphatidylethanolamine, and phosphatidylcholine; these metabolites are mainly involved in “glycerolipid metabolism” (ko00561) and “glycerophospholipid metabolism” (ko00564), suggesting that greater lipid metabolism and transport in the rumen of HH dairy buffaloes provide more precursors for milk fat synthesis. In addition, the rumen of HH dairy buffaloes had higher concentrations of several coenzyme vitamins and volatile fatty acids, confirming our rumen metagenome view that these higher concentrations are beneficial in enhancing lactation performance. In addition, the rumen of HH dairy buffaloes had higher concentrations of several coenzyme vitamins and volatile fatty acids, confirming our rumen metagenome view that these higher concentrations are beneficial in enhancing lactation performance. Finally, Spearman’s correlation results showed that the metabolites that positively correlated with milk yield and milk fat yield were mainly lipids, carbohydrates, and organic acids, suggesting that the functions and pathways involved in these metabolites provide more metabolic energy to mammary glands of the HH dairy buffaloes via bloodstream (Wu et al., 2018). This study detected and analyzed a full class of endogenous small molecules using liquid chromatography, and future studies should employ more advanced metabolomics techniques to analyze a greater number of large metabolites and comprehensive targeted assays for key types of metabolites to further explore the impact of microbial metabolic changes on host animals.

In the serum metabolome, the metabolites that were in higher concentrations in the serum of HH dairy buffaloes compared with the LL dairy buffaloes were mainly lipids, and a small proportion of organic acids, bile acids, and heterocyclic compounds. First, in addition to the known lipid metabolites in the rumen metabolome, the serum metabolome also includes phosphatidylserine, sphingomyelin, and ceramide, and these metabolites are mainly involved in “phospholipase D signaling pathway” (ko04072), “sphingolipid signaling pathway” (ko04071), and “phosphatidylinositol signaling pathway” (ko04070), suggesting that HH dairy buffaloes perform better in cellular signaling and lipid transport, which may be more conducive to transporting precursors to mammary tissue and improving the efficiency of milk fat synthesis. Second, the metabolic pathways of polyunsaturated fatty acids and vitamins were enriched in the serum of HH dairy buffaloes, suggesting a connection between rumen and host serum metabolism, which could contribute to the improvement of host metabolic capacity. Finally, Spearman’s correlation results showed that the metabolites that positively correlated with milk yield and milk fat yield were mainly lipids and organic acids, suggesting that the functions and pathways involved in these metabolites promote the formation of a stable metabolic system in HH dairy buffaloes, contributing to blood circulation, nutrient transport, and absorption. While the rumen plays a crucial role in the digestive system of ruminants, it is important to note that the gut also plays a vital part in the system (Mou, 2017). which may be worth studying together in the future to further explore the factors that influence host metabolism.

In ruminant studies, the relationship between the rumen microbiome and the metabolome has been reported for both goats (Mao et al., 2016) and dairy cows (Xue et al., 2020). However, whether and how the rumen microbes could interact with the microbial metabolites and host metabolites remains unknown. Our current study demonstrates a positive correlation between specific microbiota and both rumen phenotype-associated metabolites and serum phenotype-associated metabolites. By Spearman’s correlation analysis, we found that rumen phenotype-associated metabolites showed a strong positive correlation mainly with several species *Prevotella*, *Butyrivibrio*, *Barnesiella*, and *Bacteroides*, while serum phenotype-associated metabolites showed a strong positive correlation mainly with several species *Prevotella*, *Bacteroides*, *Barnesiella*, *Ruminococcus*, and *Butyrivibrio*. Additionally, the phenotype-associated metabolites in both the rumen and serum were also significantly correlated with the 17 KEGG functional modules of the rumen microbiome, suggesting a responsive relationship between these metabolites and the functions of the rumen microbiome. In summary, the composition and functions of rumen microbes affect both microbial and host metabolism, which, in turn, impact host milk production traits. Specifically, 8 species have been identified as having a significant impact on these processes, including *Prevotella.*sp.*CAG1031*, *Prevotella.*sp.*HUN102*, *Prevotella.*sp.*KHD1*, *Prevotella.phocaeensis*, *Butyrivibrio.*sp.*AE3009*, *Barnesiella.*sp.*An22*, *Bacteroides.*sp.*CAG927*, and *Bacteroidales.bacterium.52–46*, which are closely associated with phenotype-associated metabolites in the rumen and serum, may be a key contributor to high milk yield and milk fat yield in HH dairy buffaloes, the role of these microorganisms can be further validated in the future through colony transplantation. These findings provide new insights into the interactions among ruminant rumen microbial composition, functions, metabolism, and host metabolism, and the functional mechanisms by which they collectively contribute to altered host animal traits.

Recent studies have reported that the host, together with the rumen microbiome, affect dairy cow traits, including methane production (Difford et al., 2018), feed conversion efficiency (Xue et al., 2022), and milk production traits (Wallace et al., 2019). Our current findings suggest that the rumen microbiome, rumen metabolome, and serum metabolome influence host milk production and milk fat yield. Meanwhile, we refer to a study on rumen microbiome affects milk protein yield in dairy cows (Xue et al., 2020), then calculated the proportional effects of rumen microbial composition, functions, metabolism, and host metabolism on the variation in milk yield and milk fat yield were 34.04, 47.13, 39.09, and 50.14%, respectively, defined as “omics-explainability.” This concept was first proposed by Difford et al. in dairy cows, estimating by quantifying the cumulative effect of microbial abundance on the variation in phenotypic traits. This concept has been applied to studies on several animals, including pigs (Camarinha-Silva et al., 2017), chickens (Wen et al., 2019), and dairy cows (Difford et al., 2018) but has not yet been reported on dairy buffaloes. Our study suggests that host serum metabolism had the greatest influence on milk yield and milk fat yield in dairy buffaloes, followed by the functions of rumen microorganisms. Although several studies have indicated that rumen microbial composition and functions significantly contribute to the different individualized performance of ruminants, such as feed conversion efficiency (Li and Guan, 2017) and methane emissions (Difford et al., 2018). However, it is suggested by our results that host metabolism is a crucial factor that cannot be overlooked in future studies and aimed at enhancing feed conversion efficiency and production performance in ruminants, even better prediction of milk production traits may be achieved by utilizing host serum metabolites. Compared with models that use only host animal genetic data, further research testing the predictive accuracy of multi-omics data for milk production traits will provide more evidence for this potential new selection criterion.

Although the factors affecting the milk yield and milk fat yield of dairy buffaloes including feeding environment, diet, feeding management, age, parity, lactation stage, and breed were largely controlled in our study, we found that variations in milk production traits were also attributed to variations in rumen microorganisms and their metabolites, as well as the host’s utilization and absorption of metabolites. In addition to the aforementioned factors, this milk production traits could also be attributed to variations in genetics. Recent studies have reported that genetic factors not only affect the phenotypic characteristics of ruminants but also the rumen microbiota, and the heritable microbial taxa were associated with feed efficiency and methane emission (Abbas et al., 2019), as well as the possible existence of different heritability for those microbes, functions, and metabolites (Li et al., 2019). Further studies are required to assess this heritability, providing more evidence for the possibility of manipulating rumen microbes, functions, and metabolites through genetic selection.

## Conclusion

Our research has identified the rumen microbial taxonomic features, functions, and metabolites together with their interactions with host metabolism that contribute to milk yield and milk fat yield in dairy buffaloes. Dairy buffaloes with higher milk yield and milk fat yield had lower abundances of methanogenic archaea and methanogenic functions, leading to higher functions and enzymes involved in carbohydrate synthesis and higher concentrations of VFAs in the rumen. The microorganisms in the rumen of HH dairy buffaloes serve as stronger vitamin B producers, meeting the requirement for higher milk production performances. Mainly eight species were enriched in the rumen of HH dairy buffaloes and were closely associated with fatty acid biosynthesis functions, lipid metabolism functions, rumen lipid metabolites, and serum lipid metabolites, including *Prevotella.*sp.*CAG1031*, *Prevotella.*sp.*HUN102*, *Prevotella.*sp.*KHD1*, *Prevotella.phocaeensis*, *Butyrivibrio.*sp.*AE3009*, *Barnesiella.*sp.*An22*, *Bacteroides.*sp.*CAG927*, and *Bacteroidales.bacterium.52–46*, which provide more precursors for milk fat synthesis. As the outcome of the microbial composition and functions differences, we found higher concentrations of metabolites (mainly lipids, carbohydrates, and organic acids) and end-products of VFAs (mainly acetic and butyric acids) in the rumen of HH dairy buffaloes, as well as higher concentrations of metabolites (mainly lipids and organic acids) in the serum of HH dairy buffaloes, suggesting that variations in rumen microbial metabolism contribute to differences in metabolites that are absorbed and transported by the host. The “omics-explainability” results indicated that serum metabolites and rumen microbial functions had a greater impact on milk yield and milk fat yield than rumen microbial metabolites and microbial composition. In conclusion, these findings provide insights into strategies for modifying the rumen microbiota for higher yield and quality of buffalo milk through feeding management, colony transplantation, or genetic selection.

## Materials and methods

### Animals, sampling, and physiological parameters measurement

#### Animal phenotype and feeding management

Based on complete phenotypic data (milk production and milk composition) obtained from 24 weeks of observation, collection, testing, and recording, 12 high-yield dairy buffaloes (dairy buffaloes with high milk yield and milk fat yield; HH group) and 12 low-yield dairy buffaloes (dairy buffaloes with low milk yield and milk fat yield; LL group) were selected from the cohort of 226 healthy mid-lactation Murrah dairy buffaloes at a local commercial dairy buffalo breeding base in Guangxi. Dairy buffaloes received the same diet with a concentrate-to-forage ratio of 30:70 (dry matter basis).

#### Collection of rumen contents

After 4 h of morning feeding, rumen contents were sampled using oral stomach tubes and preserved in liquid nitrogen; VFAs contents were measured using an Agilent 7890B-7000D GC–MS/MS.

#### Collection of blood

Using anticoagulant blood collection vessels to collect blood from the jugular vein and preserve in liquid nitrogen, biochemical indicators in the blood were measured using a URIT automated biochemistry analyzer CA-810B.

### Analysis of rumen metagenome

#### DNA extraction and detection

Total genomic DNA was extracted from rumen contents using the TIANGEN Magnetic Universal Genomic DNA Kit. DNA purity and integrity were analyzed using 1% agarose gel electrophoresis (AGE). DNA was quantified using the Qubit® dsDNA Assay Kit in Qubit® 2.0 Fluorometer (Life Technologies, CA, USA). The DNA sample is diluted with sterile water until the OD value is between 1.8 and 2.0.

#### Library construction and sequencing

Taking 1 μg of DNA sample, library construction was performed using the NEBNext® Ultra™ DNA Library Prep Kit for Illumina® (NEB, USA). The qualified DNA samples were randomly broken into fragments of approximately 350 bp by Covaris ultrasonic crusher, and the whole library preparation was completed through the steps of end repair, addition of A-tail, addition of sequencing connector, purification, and PCR amplification. After the library was constructed, Qubit 2.0 was used for preliminary quantification, and the library was diluted to 2 ng/μL. Then, the insert size of the library was detected by Agilent 2,100, and after the insert size conformed to the expectation, Q-PCR was used to accurately quantify the effective concentration of the library (the effective concentration of the library was >3 nM), so as to ensure the quality of the library. The quality of the library was ensured by the Q-PCR method. After passing the library inspection, different libraries are pooled according to the effective concentration and the target downstream data volume and then sequenced by Illumina PE150.

#### Sequencing results pretreatment

Preprocessing the Raw Data obtained from the Illumina HiSeq sequencing platform using Readfq (V8^1^) was conducted to acquire the Clean Data for subsequent analysis: Removes reads that contain more than a certain percentage of low-quality bases (quality value ≤38) (set to 40 bp by default). Remove N bases up to a certain percentage of reads (default set to 10 bp). Removes reads that overlap with the Adapter above a certain threshold (set to 15 bp by default). If there is host contamination in the sample, it needs to be compared with the host sequence to filter out reads that may originate from the host (Karlsson et al., 2012, 2013; Scher et al., 2013) (Bowtie2 software is used by default with the parameter settings: -end-to-end, −sensitive, -I 200, −X 400^2^).

#### Metagenome assembly

After preprocessing to get Clean Data, MEGAHIT assembly software (v1.0.4-beta) was used for assembly analysis (Assembly Analysis), assembly parameters: -presets meta-large (−-min-count 2 --k-min 27 --k-max 87 --k-step 10). Scaffolds obtained from assembly are broken from N junctions to obtain N-free sequence fragments called Scaftigs (Qin et al., 2010; Li et al., 2015) (i.e., continuous sequences within scaffolds). After QC of each sample, CleanData was compared with the assembled Scaftigs of each sample using Bowtie2 software with the comparison parameters (Karlsson et al., 2013; Nielsen et al., 2014): -end-to-end, −sensitive, -I 200, and -X 400. For Scaftigs generated from single-sample assembly, fragments below 500 bp (Karlsson et al., 2013; Li et al., 2014; Zeller et al., 2014; Sunagawa et al., 2015) were filtered out and subjected to statistical analysis and subsequent gene prediction.

#### Gene prediction and abundance analysis

Open Reading Frame (ORF) prediction was performed using MetaGeneMark software (V2.10^3^) (Karlsson et al., 2012; Mende et al., 2012; Li et al., 2014; Oh et al., 2014; Qin et al., 2014) from the Scaftigs (≥ 500 bp) of each sample, and from the prediction results, the information with length of less than 100 nt (Qin et al., 2010; Zhu et al., 2010; Nielsen et al., 2014; Zeller et al., 2014; Sunagawa et al., 2015) was filtered out. The ORF prediction results of each sample assembly were de-redundant using CD-HIT software (V4.5.8^4^) (Li and Godzik, 2006; Fu et al., 2012), to obtain non-redundant initial gene catalog (here, operationally, non-redundant sequences of nucleic acids encoding consecutive genes are referred to as genes (Zeller et al., 2014)), which were clustered by default with identity 95% and coverage 90% (Li et al., 2014; Qin et al., 2014), and the longest sequences were selected as representative sequences using the parameters: -c 0.95, −G 0, -aS 0.9, −g 1, −d 0. Using Bowtie2, Clean Data of each sample was compared with the initial gene catalog, and the number of reads on the comparison of the gene in each sample was calculated to obtain the number of reads on the comparison with the comparison parameters (Qin et al., 2010; Li et al., 2014): -end-to-end, −sensitive, -I 200, and -X 400. Genes that supported a read count of ≤2 (Zeller et al., 2014) in individual samples were filtered out to obtain the final gene catalog (Unigenes) for subsequent analysis. From the number of reads on the comparison and the gene length, the abundance information of each gene in each sample was calculated (Cotillard et al., 2013; Le Chatelier et al., 2013; Villar et al., 2015). Based on the abundance information of each gene in the gene catalog in each sample, downstream analysis was performed.

#### Taxonomy prediction

Using DIAMOND software, (V0.9.9^5^) (Buchfink et al., 2015) the Unigenes were compared to bacterial, fungal, archaeal, and viral sequences drawn from NCBI’s NR (Version: 2018-01-02^6^) database (blastp, evalue ≤1e-5) (Karlsson et al., 2013). Filtering of results: For each sequence, the results with evalue ≤ min evalue*10 are selected for subsequent analysis. After filtering, since each sequence may have multiple comparisons and get multiple different species classification information, in order to ensure its biological significance, the LCA algorithm (applied to the systematic classification of the MEGAN (Huson et al., 2011) software) is adopted, and prior to the appearance of the first branch, the taxonomic level is used as the species annotation information for the sequence. Starting from the LCA annotation results and gene abundance tables, information on the abundance of individual samples at each taxonomic level (phylum, order, family, genus, and species) is obtained, and for a given species, the abundance in a given sample is equal to the sum of the abundance of the genes annotated to that species (Karlsson et al., 2012; Li et al., 2014; Feng et al., 2015). From the LCA annotation results and gene abundance tables, a table of the number of genes in each sample at each taxonomic level (phylum, order, family, genus, and species) was obtained, and the number of genes in a given sample for a given species was equal to the number of genes whose abundance was not zero among the genes annotated to that species. Subsequent analysis was conducted from abundance tables at each taxonomic level (kingdom, phylum, order, family, genus, and species).

#### Common functional database annotations

Unigenes were compared with each functional database using DIAMOND software (blastp, evalue ≤1e-5) (Li et al., 2014; Feng et al., 2015). Result filtering: For each sequence, the result with the highest score (one HSP > 60 bits) is selected for subsequent analysis (Qin et al., 2012, 2014; Li et al., 2014; Backhed et al., 2015). From the comparison results, the relative abundance of different functional tiers [the relative abundance of each functional tier is equal to the sum of the relative abundance of the genes annotated to that functional tier (Karlsson et al., 2012; Li et al., 2014)] was counted, in which the KEGG (Kanehisa et al., 2006, 2017) database (version 2018-01-01^7^) was divided into 6 tiers: level1, level2, level3, KO, ec, and module, and the CAZy (Cantarel et al., 2009) database (version 2018–01^8^) was divided into 3 tiers: level1, level2, and level3. From the functional annotation results and the gene abundance tables, a table of the number of genes in each sample at each taxonomic level was obtained, and for a given function, the number of genes in a given sample was equal to the number of genes whose abundance was not 0 among the genes annotated for that function. Based on the abundance table of each taxonomy hierarchy, the counting of annotated gene numbers, the exhibition of the general relative abundance situation, and subsequent analysis.

### Analysis of rumen and serum metabolome

#### Sample pretreatment of rumen contents

##### Methods for extraction of hydrophilic compounds

The sample was taken out from the −80°C refrigerator and thawed on ice and vortexed for 10 s. In total, 200 μL of sample and 200 μL of 20% acetonitrile methanol internal standard extractant were mixed. The mixture was vortexed for 3 min and centrifuged (12,000 rpm, 4°C) for 10 min. Then, 350 μL of the supernatant was transferred and dried. The dry residue was reconstituted with 150 μL of 70% methanol water, vortexed for 3 min, and sonicated for 10 min in ice water bath. Finally, the supernatant was centrifuged (12,000 rpm, 4°C) for 3 min and then analyzed.

##### Methods for extraction of hydrophobic compounds

The sample was taken out from the −80°C refrigerator, thawed on ice, and vortexed for 10 s. In total, 200 μL of the sample and 1 mL of the extraction solvent (MTBE: MeOH = 3:1, v/v) were mixed containing internal standard mixture. After whirling the mixture for 15 min, 100 μL of water was added, vortexed for 1 min, and then centrifuged at 12,000 rpm for 10 min. In total, 500 μL of the upper organic layer was collected and evaporated using a vacuum concentrator. The dry extract was reconstituted using 200 μL mobile phase B prior to LC–MS/MS analysis.

#### Sample pretreatment of serum

##### Methods for extraction of hydrophilic compounds

The sample was taken out from the −80°C refrigerator, thawed on ice, and vortexed for 10 s. In total, 50 μL of sample and 300 μL of 20% acetonitrile methanol internal standard extractant were mixed. The mixture was vortexed for 3 min and centrifuged (12,000 rpm, 4°C) for 10 min. Then, 200 μL of the supernatant was transferred and stored at −20°C for 30 min. Finally, the supernatant was centrifuged (12,000 rpm, 4°C) for 3 min and then analyzed.

##### Methods for extraction of hydrophobic compounds

The sample was taken out from the −80°C refrigerator, thawed on ice, and vortexed for 10 s. In total, 50 μL of the sample and 1 mL of the extraction solvent (MTBE: MeOH = 3:1, v/v) were mixed containing internal standard mixture. After whirling the mixture for 15 min, 200 μL of water was added, vortexed for 1 min, and then centrifuged at 12,000 rpm for 10 min. Overall, 200 μL of the upper organic layer was collected and evaporated using a vacuum concentrator. The dry extract was reconstituted using 200 μL mobile phase B prior to LC–MS/MS analysis.

#### UPLC conditions

##### UPLC conditions of hydrophilic compounds

T3 UPLC Conditions: The sample extracts were analyzed using an LC-ESI-MS/MS system (UPLC, ExionLC AD^9^; MS, QTRAP® System^10^). The analytical conditions were as follows, UPLC: column, Waters ACQUITY UPLC HSS T3 C18 (1.8 μm, 2.1 mm*100 mm); column temperature, 40°C; flow rate, 0.4 mL/min; injection volume, 2 μL; solvent system, water (0.1% formic acid): acetonitrile (0.1% formic acid); gradient program, 95:5 V/V at 0 min, 10:90 V/V at 11.0 min, 10:90 V/V at 12.0 min, 95:5 V/V at 12.1 min, 95:5 V/V at 14.0 min.

Hilic Amide UPLC Conditions: The sample extracts were analyzed using an LC-ESI-MS/MS system [UPLC, ExionLC AD (see Footnote 9); MS, QTRAP® System (see Footnote 10)]. The analytical conditions were as follows: UPLC: column, Waters ACQUITY UPLC BEH Amide (1.7 μm, 2.1 mm*100 mm); Column temperature, 40°C; Flow rate, 0.4 mL/min; Injection volume, 2 μL; Solvent system, water (20 mM Ammonium formate and 0.4% ammonia): acetonitrile; Gradient program, 10:90 V/V at 0 min, 40:60 V/V at 9.0 min, 60:40 V/V at 10.0 min, 60:40 V/V at 11.0 min, 10:90 V/V at 11.1 min, and 10:90 V/V at 15.0 min.

##### UPLC conditions of hydrophobic compounds

The sample extracts were analyzed using an LC-ESI-MS/MS system [UPLC, ExionLC AD (see Footnote 9); MS, QTRAP® System (see Footnote 10)]. The analytical conditions were as follows, UPLC: column, Thermo Accucore™ C30 (2.6 μm, 2.1 mm*100 mm i.d.); solvent system, A: acetonitrile/water (60/40 V/V, 0.1% formic acid, 10 mmol/L ammonium formate). B: acetonitrile/isopropanol (10/90 V/V, 0.1% formic acid, 10 mmol/L ammonium formate); gradient program, A/B (80:20 V/V) at 0 min, 70:30 V/V at 2.0 min, 40:60 V/V at 4 min, 15:85 V/V at 9 min, 10:90 V/V at 14 min, 5:95 V/V at 15.5 min, 5:95 V/V at 17.3 min, 80:20 V/V at 17.3 min, 80:20 V/V at 20 min; flow rate, 0.35 mL/min; temperature, 45°C; injection volume: 2 μL. The effluent was alternatively connected to an ESI-triple quadrupole-linear ion trap (QTRAP)-MS.

#### ESI-Q TRAP-MS/MS

##### ESI-Q TRAP-MS/MS of hydrophilic compounds

T3 and Hilic Amide have the same mass spectrometry parameters. LIT and triple quadrupole (QQQ) scans were acquired on a triple quadrupole-linear ion trap mass spectrometer (QTRAP), QTRAP® LC–MS/MS System, equipped with an ESI Turbo Ion-Spray interface, operating in positive and negative ion mode and controlled by Analyst 1.6.3 software (Sciex). The ESI source operation parameters were as follows: source temperature 500°C; ion spray voltage (IS) 5,500 V (positive), −4,500 V (negative); ion source gas I (GSI), gas II (GSII), curtain gas (CUR) were set at 55, 60, and 25.0 psi, respectively. The collision gas (CAD) was high. Instrument tuning and mass calibration were performed with 10 and 100 μmol/L polypropylene glycol solutions in QQQ and LIT modes, respectively. A specific set of MRM transitions were monitored for each period, according to the metabolites eluted within this period.

##### ESI-Q TRAP-MS/MS of hydrophobic compounds

LIT and triple quadrupole (QQQ) scans were acquired on a triple quadrupole-linear ion trap mass spectrometer (QTRAP), QTRAP® LC–MS/MS System, equipped with an ESI Turbo Ion-Spray interface, operating in positive and negative ion mode and controlled by Analyst 1.6.3 software (Sciex). The ESI source operation parameters were as follows: ion source, turbo spray; source temperature 500°C; ion spray voltage (IS) 5,500 V (positive), −4,500 V (neagtive); ion source gas 1 (GS1), gas 2 (GS2), curtain gas (CUR) were set at 45, 55, and 35 psi, respectively; The collision gas (CAD) was medium. Instrument tuning and mass calibration were performed with 10 and 100 μmol/L polypropylene glycol solutions in QQQ and LIT modes, respectively. QQQ scans were acquired as MRM experiments with collision gas (nitrogen) set to 5 psi. DP and CE for individual MRM transitions were performed with further DP and CE optimization. A specific set of MRM transitions were monitored for each period, according to the metabolites eluted within this period.

##### KEGG annotation and enrichment analysis

Identified metabolites were annotated using the KEGG compound database^11^; annotated metabolites were then mapped to the KEGG pathway database.^12^ Significantly enriched pathways are identified with a value of *p* of hypergeometric test for a given list of metabolites.

### Construction of rumen microbial, rumen microbial functional, rumen metabolic, and serum metabolic relationship matrix

The relative abundances of rumen microbial composition (species-level microbial), rumen microbial function (KOs), rumen metabolome (rumen metabolites), and serum metabolome (serum metabolites) were subjected to z-score processing and were normalized to have a zero mean and a unit variance and then were used to construct the matrix M, K, R, and S, respectively (Difford et al., 2018; Xue et al., 2020). A linear mixed model was constructed for phenotype (milk yield and milk fat yield), and the linear mixed model utilized to estimate the variances of four omics was calculated as follows:


$$y_{ijk=\mu +p_{j}+d_{k}+m_{i}+e_{ijk}}$$

$$y_{ijk=\mu +p_{j}+d_{k}+k_{i}+e_{ijk}}$$

$$y_{ijk=\mu +p_{j}+d_{k}+r_{i}+e_{ijk}}$$

$$y_{ijk=\mu +p_{j}+d_{k}+s_{i}+e_{ijk}}$$

where $y_{ijk}$ is the phenotype (milk yield and milk fat yield) (kg/day); μ is the model intercept; $p_{j}$ is the parity fixed effect; $d_{k}$ is the lactation days fixed effect; $m_{i}$ is the rumen microbial random effect for the $i\mathrm{th}\;\mathrm{animal}\sim \mathrm{NID}\left(0\mathrm{,M}\sigma_{m}^{2}\right)$ , where $\sigma_{m}^{2}$ is the rumen microbial variance and M is the microbial relationship matrix; and $e_{ijk}$ is the residual effects. $k_{i}$ is the rumen microbial function random effect for the $i\mathrm{th}\;\mathrm{animal}\sim \mathrm{NID}\left(0\mathrm{,K}\sigma_{k}^{2}\right)$ . $r_{i}$ is the rumen metabolites random effect for the $i\mathrm{th}\;\mathrm{animal}\sim \mathrm{NID}\left(0\mathrm{,R}\sigma_{r}^{2}\right)$ . $s_{i}$ is the serum metabolites random effect for the $i\mathrm{th}\;\mathrm{animal}\sim \mathrm{NID}\left(0\mathrm{,S}\sigma_{s}^{2}\right)$ . The phenotypic variance that explained by the rumen microbial variance, rumen microbial functional variance, rumen metabolic variance, and serum metabolic variance was estimated as $\frac{\sigma_{m}^{2}}{\sigma_{p}^{2}}$ , $\frac{\sigma_{k}^{2}}{\sigma_{p}^{2}}$ , $\frac{\sigma_{r}^{2}}{\sigma_{p}^{2}}$ , and $\frac{\sigma_{s}^{2}}{\sigma_{p}^{2}}$ , respectively, where $\sigma_{p}^{2}$ is the phenotypic (milk yield and milk fat yield) variance. The linear mixed model was performed using the “lme4” package in R.^13^

### Statistical analysis

Milk production traits, rumen VFA concentrations, blood routine parameters, and serum biochemical parameters were compared using t-tests, and *p*-value < 0.05 was considered as significant.

In rumen metagenome, Krona analysis was used to visualize the species annotation results (Qin et al., 2012). A bar chart was used to show the relative abundance of species annotation results at domain, phylum, genus and species levels. The difference between groups is tested by Adonis analysis (R vegan package, version 2.15.3); we performed the permutation multivariate analysis of variance (PERMANOVA) on the microbial abundance profiles using microbial Bray–Curtis distance, and based on Bray–Curtis dissimilarity matrices at species level, the PCoA analysis was performed. Metastats and LEfSe analysis are used to look for the different species between groups (White et al., 2009). Permutation test between groups is used in Metastats analysis for each taxonomy and get the *p* value, then use Benjamini and Hochberg False Discovery Rate to correct *p* value, with the FDR adjusted *p* value < 0.05 being considered as significantly different. The species of the rumen microbiota were compared using LEfSe analysis (Segata et al., 2011), and LEfSe analysis is conducted by LEfSe software (the default LDA score is 2), and significant differences were examined by an LDA score of > 2 and *p*-value of < 0.05. The LEfSe analysis of functional difference between the two groups was performed, comparative analysis of metabolic pathways, modules, KEGG enzymes, and CAZymes, and significant differences were considered by an LDA score of > 2 and *p*-value < 0.05.

In rumen and serum metabolome, unsupervised PCA (principal component analysis) was performed by statistics function prcomp within R (see Footnote 13). The data were unit variance-scaled before unsupervised PCA. Significantly different metabolites between two groups were determined by VIP > 1 and FDR-adjusted *p*-value < 0.05. VIp values were extracted from OPLS-DA result, which also contain score plots and permutation plots, and generated using the R package MetaboAnalystR. The data were log-transformed ( $\log_{2}$ ) and mean-centered before OPLS-DA. To avoid overfitting, a permutation test (200 permutations) was performed. The metabolite datasets in the rumen and serum were compared between two groups and visualized using heat maps [“pheatmap” package in R (see Footnote 13)]. Significantly different metabolic pathways between two groups were determined by FDR-adjusted *p*-value < 0.05.

### Correlation analysis

Correlation analysis between rumen metabolites, serum metabolites, and phenotypes (milk yield and milk fat yield) was performed using Spearman’s rank correlation to identify the significantly phenotype-associated metabolites and was subsequently used for rumen microbiota and functional modules.

All correlation analyses were performed using Spearman’s rank correlation, *R* > 0.5 or < − 0.5 was considered as strong correlation, and *p*-value < 0.05 was considered as significant. The correlation heat map was generated using the R program “ComplexHeatmap” package (see Footnote 13).

## Data availability statement

The datasets presented in this study can be found in online repositories. The names of the repository/repositories and accession number(s) can be found at: https://www.ncbi.nlm.nih.gov/, PRJNA1012811; https://www.ebi.ac.uk/metabolights/, MTBLS8503; https://www.ebi.ac.uk/metabolights/, MTBLS8504.

## Ethics statement

This animal study was reviewed and approved by the Guangxi University Animal Care and Use Committee (Nanning, China), in strict compliance with the Regulations for the Administration of Affairs Concerning Experimental Animals (No. 588 Document of the State Council of the People’s Republic of China, 2011), and informed consent was obtained from the company’s directors to allow their animals to participate in this study. The study was conducted in accordance with the local legislation and institutional requirements.

## Author contributions

BJ: Writing – original draft, Data curation, Investigation, Validation. CQ: Writing – original draft. YX: Writing – original draft. XS: Writing – original draft. YF: Writing – original draft. RL: Writing – original draft. QL: Writing – review & editing. DS: Writing – review & editing.

## Footnotes

^1^https://github.com/cjfields/readfq

^2^http://bowtie-bio.sourceforge.net/bowtie2/index.shtml

^3^http://topaz.gatech.edu/GeneMark/

^4^http://www.bioinformatics.org/cd-hit

^5^https://github.com/bbuchfink/diamond/

^6^https://www.ncbi.nlm.nih.gov/

^7^http://www.kegg.jp/kegg/

^8^http://www.cazy.org/

^9^https://sciex.com.cn/

^10^https://sciex.com/

^11^http://www.kegg.jp/kegg/compound/

^12^http://www.kegg.jp/kegg/pathway.html

^13^https://www.r-project.org
