# Supplementary material for: Multi-omics reveals the mechanism of rumen microbiome and its metabolome together with host metabolome participating in the regulation of milk production traits in dairy buffaloes
Source: Front Microbiol. 2024 Mar 8;15:1301292. doi: 10.3389/fmicb.2024.1301292 (PMC10959287; doi:10.3389/fmicb.2024.1301292)
Supplement: Supplementary file 1 [file Table_1.DOCX]

**Table S1 Physiological parameters of HH and LL dairy buffaloes**

|  | Mean | |  |
| --- | --- | --- | --- |
| **Performance** | HH (n=12) | LL (n=12) | *P* |
| Milk yield (kg/d) | 7.94 ± 0.84 | 4.44 ± 0.46 | < 0.01 |
| Milk protein (%) | 4.51 ± 0.33 | 4.64 ± 0.40 | 0.38 |
| Milk fat (%) | 7.72 ± 0.70 | 5.71 ± 0.49 | < 0.01 |
| Lactose (%) | 4.85 ± 0.78 | 4.84 ± 0.60 | 0.97 |
| Parity | 3.67 ± 0.89 | 4.08 ± 0.90 | 0.27 |
| DIM (d) | 137.58 ± 18.34 | 129.67 ± 17.33 | 0.29 |
| Milk fat yield (kg/d) | 0.61±0.06 | 0.24 ± 0.03 | < 0.01 |
| **Serum biochemical parameters** |  |  |  |
| Glutathione U/L | 38.33 ± 7.63 | 44.66 ± 8.33 | 0.06 |
| Glutamic transaminase U/L | 132.16 ± 26.54 | 130.08 ± 22.00 | 0.83 |
| Total protein g/l | 78.65 ± 7.82 | 84.10 ± 6.94 | 0.08 |
| Direct bilirubin umol/L | 2.97 ± 0.74 | 3.66 ± 1.59 | 0.24 |
| Total bilirubin umol/L | 3.89 ± 1.00 | 4.75 ± 2.61 | 0.18 |
| Alkaline phosphatase U/L | 104.41 ± 56.90 | 118.16 ± 54.47 | 0.55 |
| Urea nitrogen mmol/L | 8.66 ± 2.21 | 10.17 ± 2.57 | 0.13 |
| Creatinine umol/L | 73.50 ± 11.75 | 79.58 ± 20.83 | 0.38 |
| Uric acid umol/L | 29.00 ± 5.80 | 30.00 ± 7.58 | 0.72 |
| Triglycerides mmol/L | 0.28 ± 0.10 | 0.23 ± 0.11 | 0.23 |
| Total cholesterol mmol/L | 2.04 ± 0.44 | 2.34 ± 0.50 | 0.14 |
| Glutamyl transferase U/L | 17.75 ± 7.16 | 19.66 ± 9.84 | 0.59 |
| Blood glucose or glucose mmol/L | 3.14 ± 0.47 | 3.19 ± 0.69 | 0.85 |
| Fe umol/L | 11.50 ± 1.37 | 11.85 ± 1.27 | 0.51 |
| Ca mmol/L | 3.29 ± 0.82 | 3.19 ± 1.28 | 0.88 |
| Mg mmol/L | 0.74 ± 0.21 | 0.64 ± 0.23 | 0.74 |
| P mmol/L | 1.66 ± 0.85 | 1.44 ± 0.76 | 0.50 |
| Indirect bilirubin umol/L | 0.91 ± 0.42 | 1.10 ± 0.57 | 0.58 |
| Hydroxybutyrate dehydrogenase U/L | 492.83 ± 120.50 | 555.33 ± 84.79 | 0.15 |
| Albumin g/l | 36.48 ± 3.98 | 33.87 ± 2.25 | 0.30 |
| VHDL mmol/L | 2.33 ± 0.83 | 2.14 ± 0.56 | 0.09 |
| VLDL mmol/L | 0.78 ± 0.17 | 0.66 ± 0.24 | 0.04 |
| **blood routine parameters** |  |  |  |
| Leukocyte count/L | 10.33 ± 1.49 | 9.41 ± 1.50 | 0.14 |
| Lymphocytes (%) | 46.00 ± 1.80 | 45.00 ± 2.69 | 0.29 |
| Monocytes (%) | 4.91 ± 0.99 | 5.25 ± 1.21 | 0.47 |
| Absolute lymphocyte values 10^9/L | 3.41 ± 1.08 | 4.41 ± 1.37 | 0.06 |
| Red blood cell count 10^12/L | 7.83 ± 1.26 | 8.16 ± 1.03 | 0.48 |
| Haemoglobin g/L | 129.83 ± 6.50 | 129.41 ± 6.00 | 0.87 |
| Red blood cell pressure (%) | 41.08 ± 3.52 | 40.41 ± 2.15 | 0.58 |
| MCH pg | 14.97 ± 1.73 | 14.75 ± 1.35 | 0.79 |
| MCHC g/L | 354.66 ± 14.58 | 347.91 ± 22.43 | 0.39 |
| Platelet count 10^9/L | 120.00 ± 10.66 | 121.41 ± 13.19 | 0.77 |

*P* Values between HH and LL cows were calculated using t test.
