# Supplementary material for: Multi-omics reveals the mechanism of rumen microbiome and its metabolome together with host metabolome participating in the regulation of milk production traits in dairy buffaloes
Source: Front Microbiol. 2024 Mar 8;15:1301292. doi: 10.3389/fmicb.2024.1301292 (PMC10959287; doi:10.3389/fmicb.2024.1301292)
Supplement: Supplementary file 2 [file Table_2.DOCX]

**Table S2 Summary of sequence data generated from rumen samples of 12 HH and 12 LL dairy buffaloes**

| **#Sample** | **InsertSize(bp)** | **RawData** | **CleanData** | **Clean_Q20** | **Clean_Q30** | **Clean_GC(%)** | **Effective(%)** |
| --- | --- | --- | --- | --- | --- | --- | --- |
| HH1 | 350 | 10,037.92 | 9,989.75 | 97.02 | 92.06 | 47.95 | 99.52 |
| LL2 | 350 | 10,725.99 | 10,651.50 | 97.08 | 92.5 | 52.87 | 99.306 |
| LL3 | 350 | 10,652.84 | 10,610.28 | 96.68 | 91.44 | 49.41 | 99.6 |
| LL4 | 350 | 11,403.90 | 11,373.32 | 97.15 | 92.33 | 49.4 | 99.732 |
| HH5 | 350 | 11,451.43 | 11,428.38 | 96.88 | 91.81 | 50.94 | 99.799 |
| HH6 | 350 | 10,384.04 | 10,344.82 | 97.08 | 92.29 | 52.47 | 99.622 |
| HH7 | 350 | 10,742.77 | 10,727.31 | 96.89 | 91.81 | 48.56 | 99.856 |
| HH8 | 350 | 11,345.66 | 11,315.44 | 96.72 | 91.33 | 48.3 | 99.734 |
| LL9 | 350 | 11,379.10 | 11,365.93 | 96.79 | 91.69 | 49.39 | 99.884 |
| LL10 | 350 | 10,085.86 | 10,061.73 | 96.86 | 91.72 | 49.65 | 99.761 |
| HH11 | 350 | 10,659.36 | 10,642.23 | 97 | 92.1 | 49.91 | 99.839 |
| LL12 | 350 | 10,344.33 | 10,217.83 | 96.91 | 91.79 | 49.35 | 98.777 |
| HH13 | 350 | 10,107.29 | 10,081.63 | 97 | 92.22 | 49.06 | 99.746 |
| HH14 | 350 | 10,653.85 | 10,590.18 | 96.94 | 92.22 | 48.92 | 99.402 |
| LL15 | 350 | 10,679.75 | 10,656.07 | 95.75 | 89.48 | 49.29 | 99.778 |
| HH16 | 350 | 10,520.16 | 10,470.61 | 96.72 | 91.73 | 49.91 | 99.529 |
| LL17 | 350 | 11,075.22 | 11,048.89 | 96.56 | 91.27 | 47.13 | 99.762 |
| LL18 | 350 | 11,697.26 | 11,634.03 | 96.75 | 91.71 | 46.8 | 99.459 |
| HH19 | 350 | 10,121.89 | 10,083.50 | 95.65 | 90.73 | 55.65 | 99.621 |
| LL20 | 350 | 10,808.34 | 10,751.02 | 97.1 | 92.28 | 50.9 | 99.47 |
| HH21 | 350 | 10,171.91 | 10,156.19 | 97.16 | 92.56 | 53.09 | 99.845 |
| LL22 | 350 | 10,559.51 | 10,540.90 | 96.97 | 92.16 | 50.2 | 99.824 |
| LL23 | 350 | 10,272.40 | 10,190.44 | 97.08 | 92.41 | 50.73 | 99.202 |
| HH24 | 350 | 11,257.13 | 11,234.76 | 97.43 | 93.15 | 48.61 | 99.801 |

| **SampleID** | **Total len.(bp)** | **Num.** | **Average len.(bp)** | **N50 Len.(bp)** | **N90 Len.(bp)** | **Max len.(bp)** |
| --- | --- | --- | --- | --- | --- | --- |
| HH1 | 569,014,028 | 635,521 | 895.35 | 871 | 544 | 143,526 |
| LL2 | 516,986,962 | 552,858 | 935.12 | 918 | 552 | 82,756 |
| LL3 | 594,781,686 | 626,455 | 949.44 | 938 | 557 | 172,984 |
| LL4 | 628,327,754 | 647,611 | 970.22 | 971 | 559 | 71,921 |
| HH5 | 511,459,362 | 517,323 | 988.67 | 987 | 560 | 114,114 |
| HH6 | 510,600,739 | 565,730 | 902.55 | 883 | 551 | 130,765 |
| HH7 | 612,203,240 | 662,265 | 924.41 | 910 | 547 | 102,215 |
| HH8 | 634,971,501 | 667,945 | 950.63 | 942 | 552 | 131,442 |
| LL9 | 655,736,487 | 687,771 | 953.42 | 946 | 551 | 147,678 |
| LL10 | 476,102,043 | 535,836 | 888.52 | 858 | 542 | 93,199 |
| HH11 | 601,526,671 | 605,411 | 993.58 | 1,004 | 560 | 163,353 |
| LL12 | 594,180,428 | 638,058 | 931.23 | 922 | 549 | 43,893 |
| HH13 | 538,386,426 | 578,185 | 931.17 | 917 | 549 | 105,530 |
| HH14 | 559,262,325 | 598,243 | 934.84 | 933 | 550 | 62,056 |
| LL15 | 605,923,325 | 630,494 | 961.03 | 958 | 559 | 68,069 |
| HH16 | 504,862,653 | 553,094 | 912.80 | 893 | 552 | 79,392 |
| LL17 | 573,928,334 | 600,889 | 955.13 | 943 | 557 | 63,337 |
| LL18 | 566,307,098 | 631,029 | 897.43 | 875 | 551 | 64,001 |
| HH19 | 416,759,051 | 446,471 | 933.45 | 917 | 552 | 66,240 |
| LL20 | 576,454,065 | 658,559 | 875.33 | 849 | 543 | 84,646 |
| HH21 | 527,841,898 | 554,755 | 951.49 | 943 | 555 | 72,582 |
| LL22 | 536,986,490 | 565,598 | 949.41 | 930 | 554 | 102,715 |
| LL23 | 534,591,218 | 586,803 | 911.02 | 886 | 545 | 103,527 |
| HH24 | 612,222,430 | 635,095 | 963.99 | 955 | 556 | 118,171 |
