# Supplementary material for: Multi-omics reveals the mechanism of rumen microbiome and its metabolome together with host metabolome participating in the regulation of milk production traits in dairy buffaloes
Source: Front Microbiol. 2024 Mar 8;15:1301292. doi: 10.3389/fmicb.2024.1301292 (PMC10959287; doi:10.3389/fmicb.2024.1301292)
Supplement: Supplementary file 3 [file Table_3.DOCX]

**Table S3 PERMANOVA (permutational multivariate analysis of variance) of three microbial domains between HH and LL dairy buffaloes**

| Taxonomy | Df | SumsOfSqs | MeanSqs | F.Model | R2 | Pr(>F) |
| --- | --- | --- | --- | --- | --- | --- |
| *Bacteria* | 1(22) | 0.023(0.042) | 0.023(0.00190909090909091) | 11.89 | 0.351(0.649) | 0.001 |
| *Archaea* | 1(22) | 0.052(0.136) | 0.052(0.00618181818181818) | 8.409 | 0.277(0.723) | 0.008 |
| *Eukaryota* | 1(22) | 0.005(0.102) | 0.005(0.00463636363636364) | 1.028 | 0.045(0.955) | 0.357 |
| *Viruses* | 1(22) | 0.011(0.216) | 0.011(0.00981818181818182) | 1.082 | 0.047(0.953) | 0.302 |
