# Supplementary material for: Multi-omics reveals the mechanism of rumen microbiome and its metabolome together with host metabolome participating in the regulation of milk production traits in dairy buffaloes
Source: Front Microbiol. 2024 Mar 8;15:1301292. doi: 10.3389/fmicb.2024.1301292 (PMC10959287; doi:10.3389/fmicb.2024.1301292)
Supplement: Supplementary file 6 [file Table_6.DOCX]

**Table S6 Calculation of omics-explainability**

| **Key** | **PhenotypeVar** | **REVar** | **Explainability** |
| --- | --- | --- | --- |
| Rumen microbial composition | 0.045363365584041665 | 0.015440078009795558 | 0.3403644727636172 |
| Rumen microbial functions | 0.045363365584041665 | 0.02137962942552502 | 0.4712972494493693 |
| rumen metabolome | 0.045363365584041665 | 0.017734031412040507 | 0.39093288568251966 |
| host serum metabolome | 0.045363365584041665 | 0.02274375374326686 | 0.5013683056899964 |

**Key:** omics dataset

**PhenotypeVar:** Phenotypic variance

**REVar:** Random effect variance

**Explanability:** omics interpretation rate
