# Supplementary material for: Multi-omics reveals the mechanism of rumen microbiome and its metabolome together with host metabolome participating in the regulation of milk production traits in dairy buffaloes
Source: Front Microbiol. 2024 Mar 8;15:1301292. doi: 10.3389/fmicb.2024.1301292 (PMC10959287; doi:10.3389/fmicb.2024.1301292)

Figure S1 Profiles of rumen microbial composition of dairy buffaloes

A. Rumen microbial composition based on the domain-level taxonomy

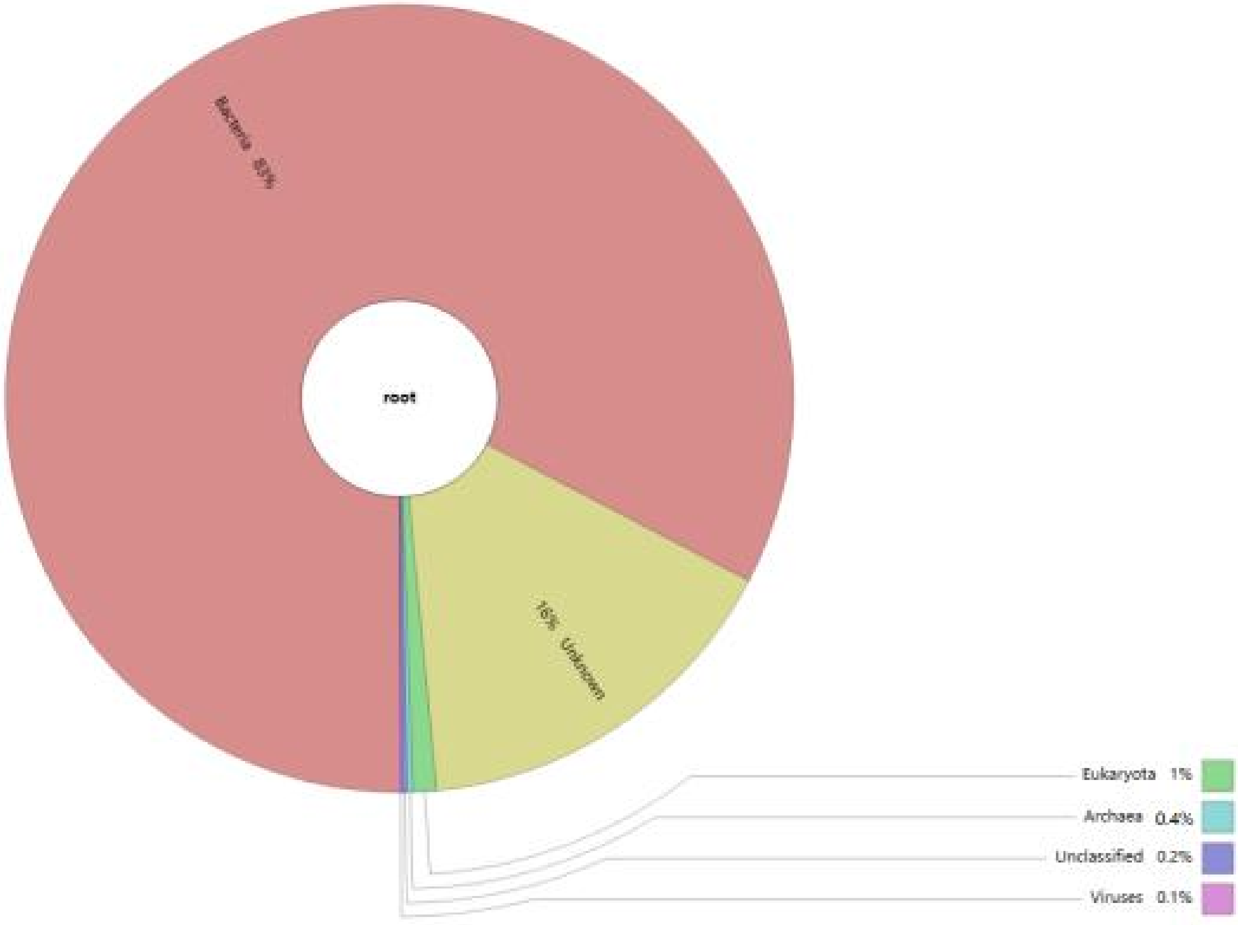

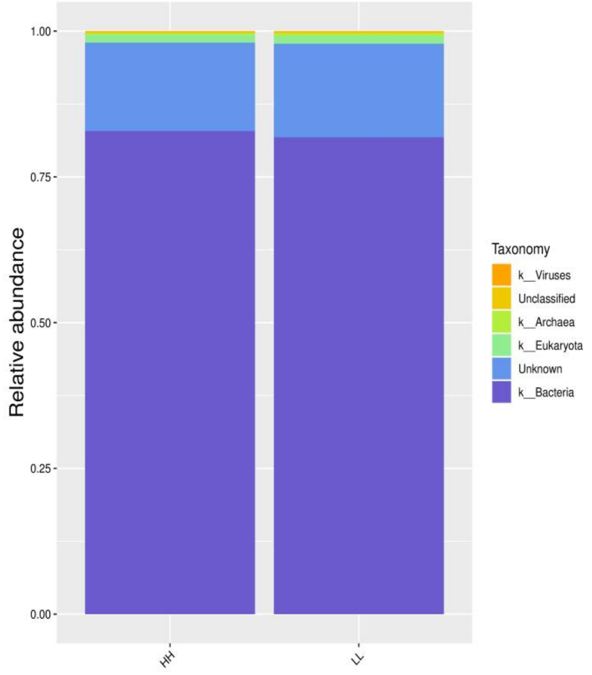

B. Bacterial composition based on the phylum-, order-, family-, genus- and species-level taxonomy.

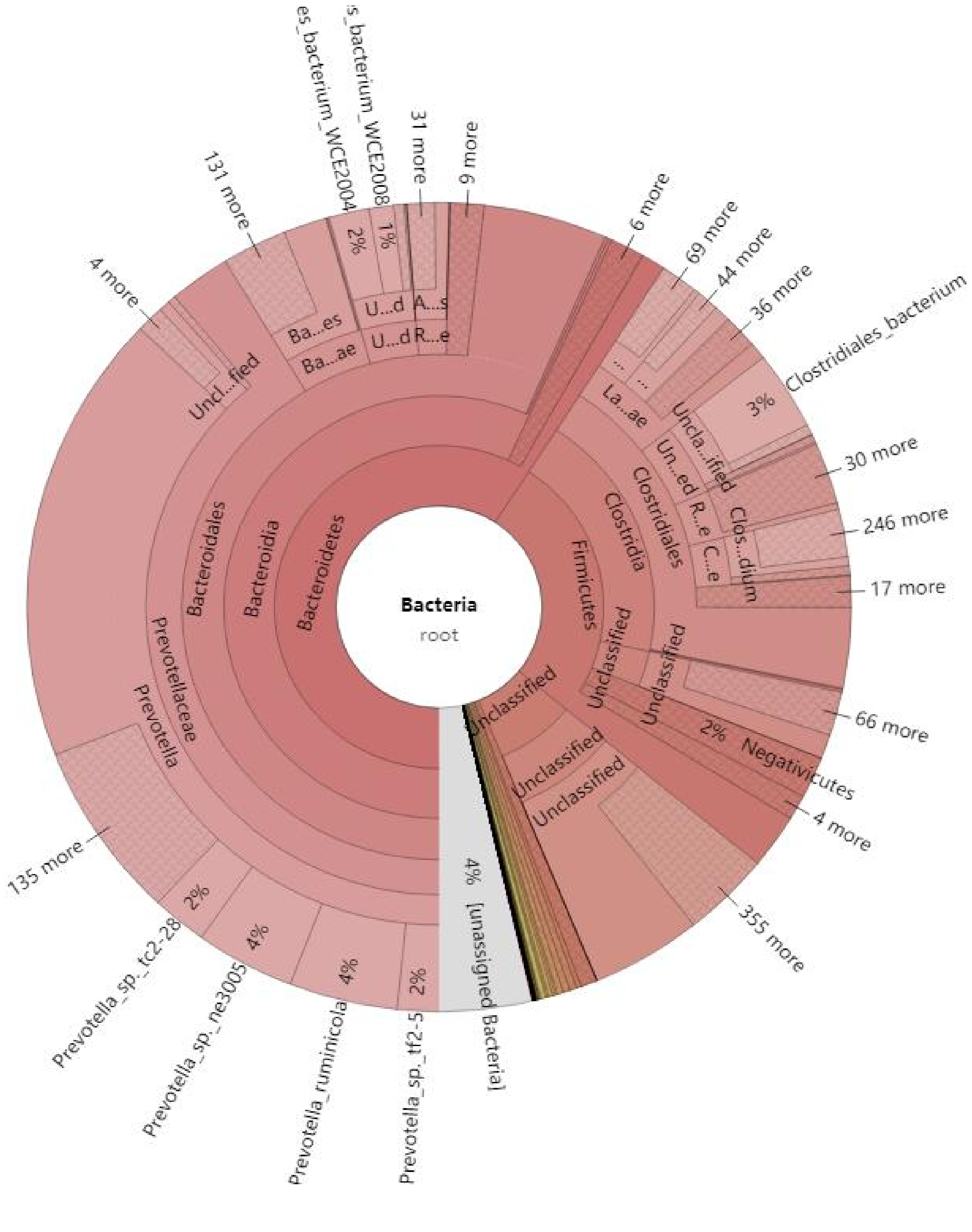

C. Archaeal composition based on the phylum-, order-, family-, genus- and species-level taxonomy.

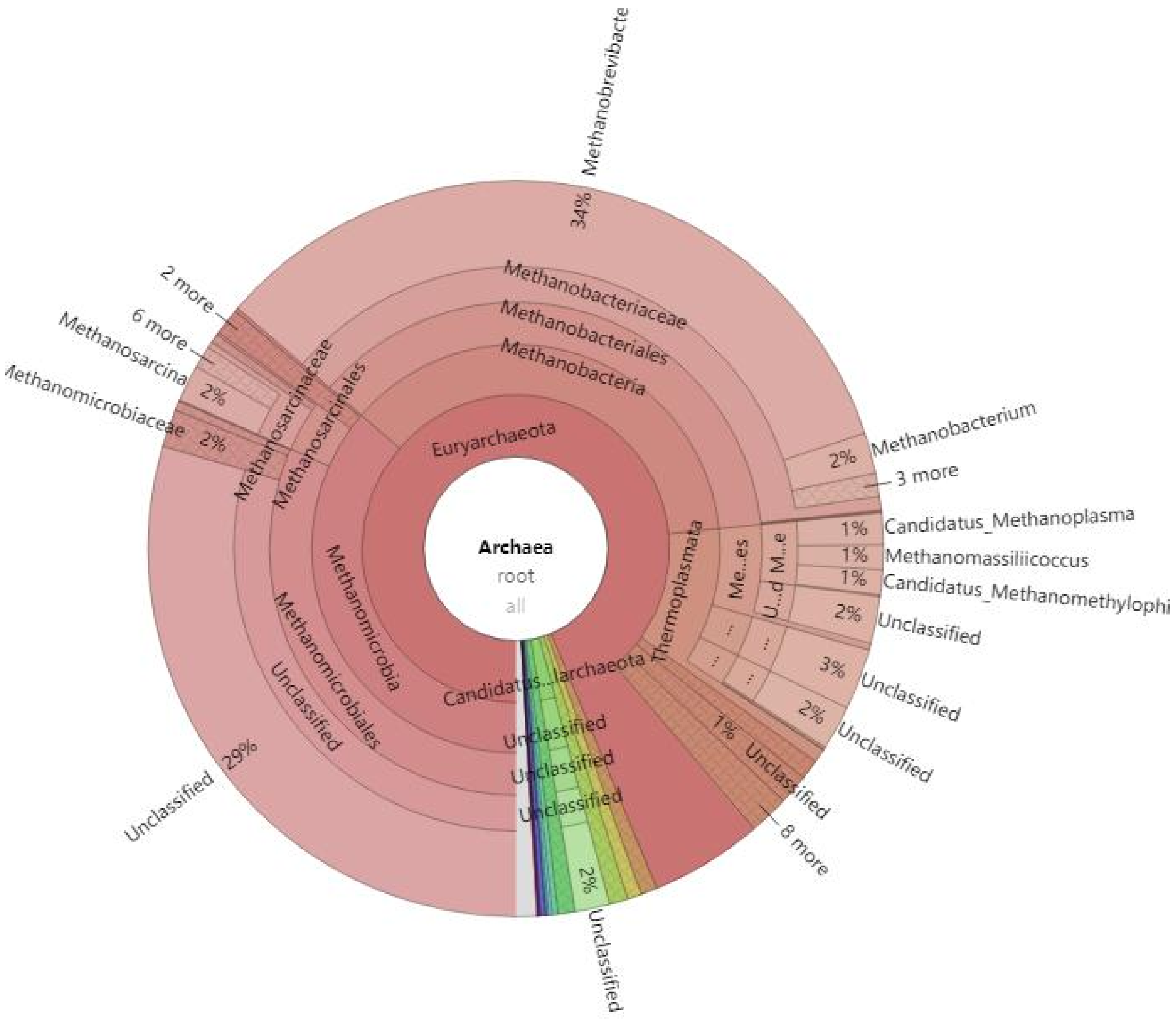

D. Viral composition based on the phylum-, order-, family-, genus- and species-level taxonomy.

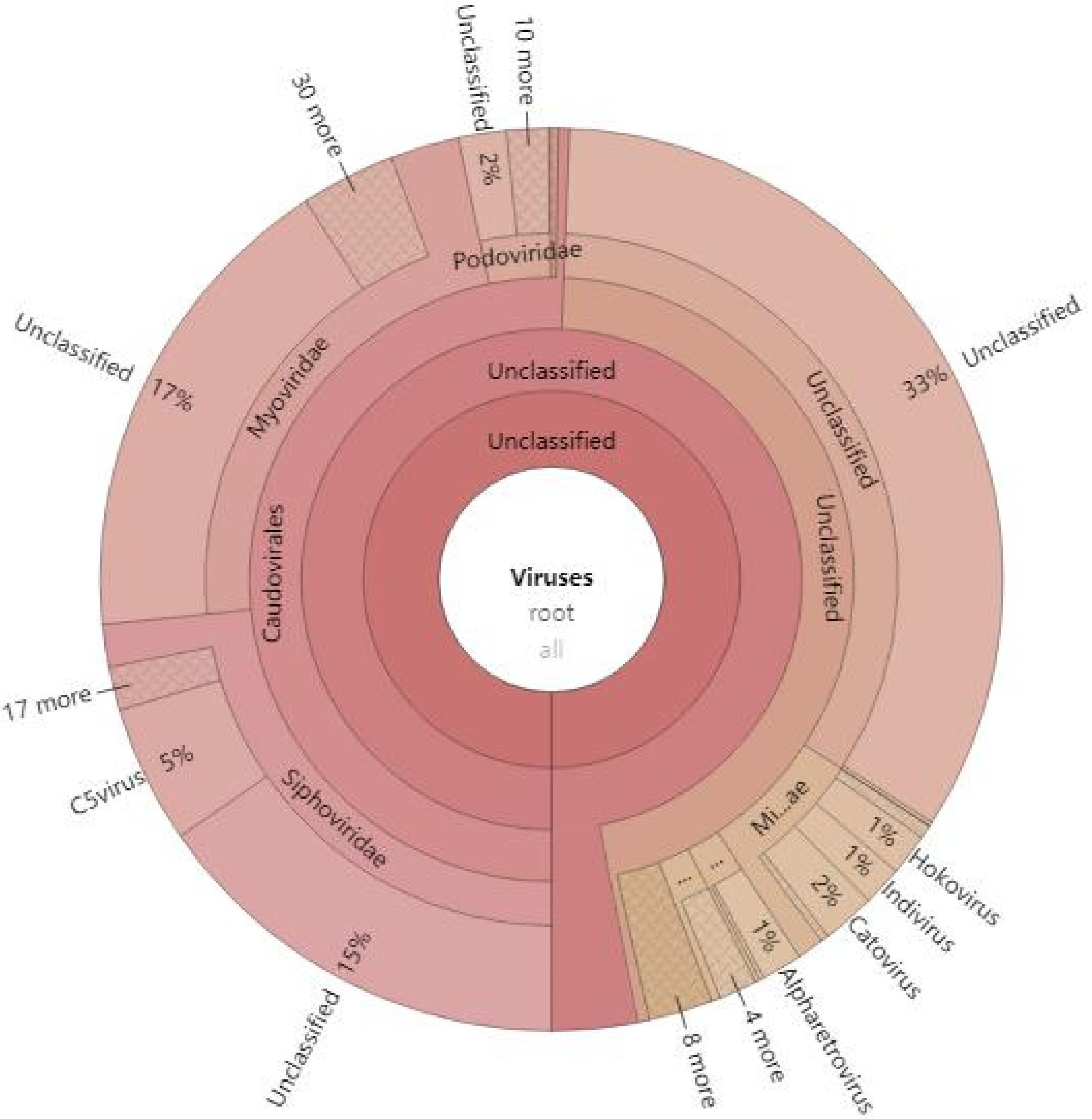

E. Eukaryotic composition based on the phylum-, order-, family-, genus- and species-level taxonomy.

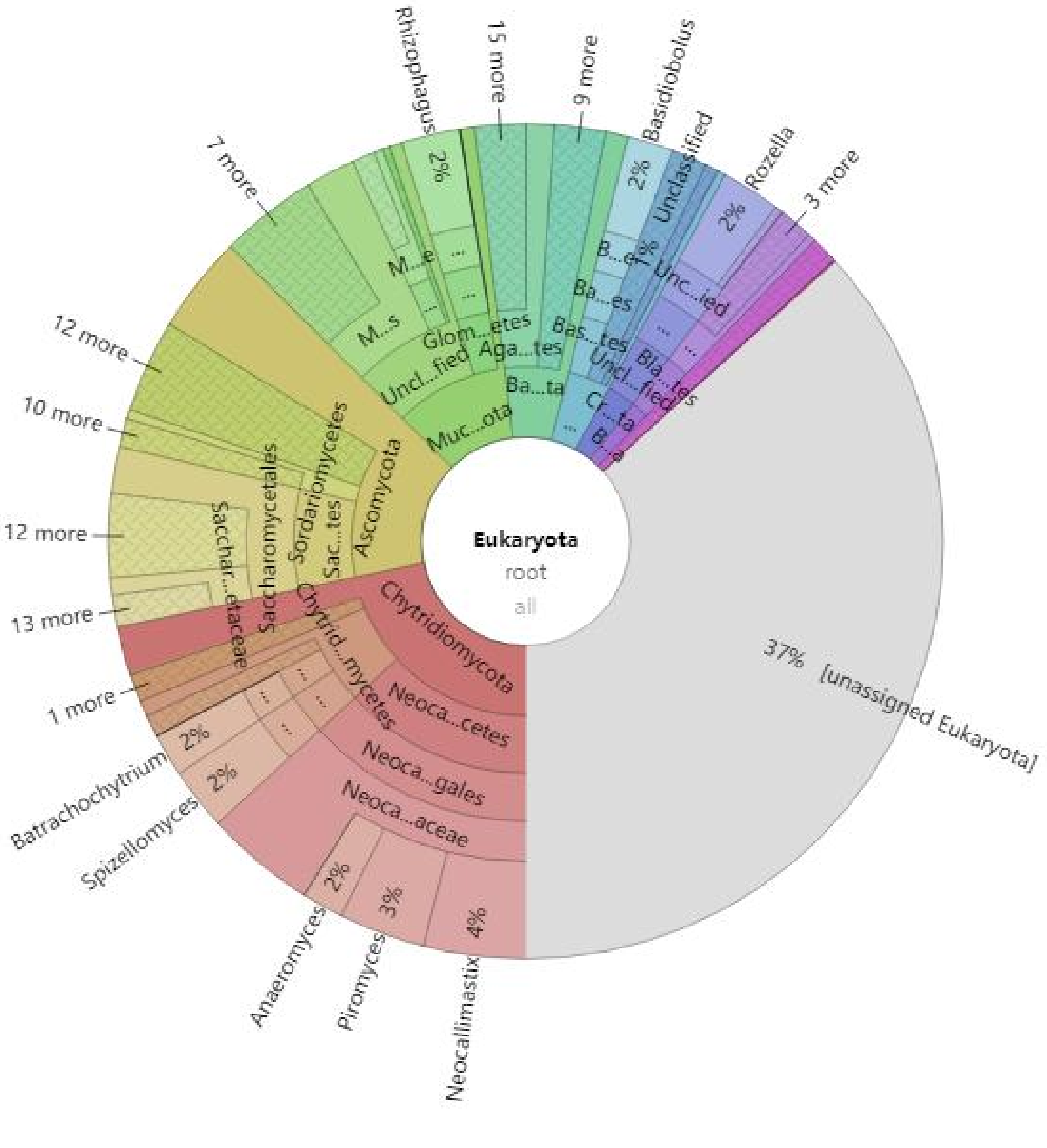

Supplement: Supplementary file 7 [file Image_1.pdf]
