# Supplementary material for: Multi-omics reveals the mechanism of rumen microbiome and its metabolome together with host metabolome participating in the regulation of milk production traits in dairy buffaloes
Source: Front Microbiol. 2024 Mar 8;15:1301292. doi: 10.3389/fmicb.2024.1301292 (PMC10959287; doi:10.3389/fmicb.2024.1301292)

Figure S2 Comparison of bacterial phyla, genera and species

A. The main dominant bacterial phyla

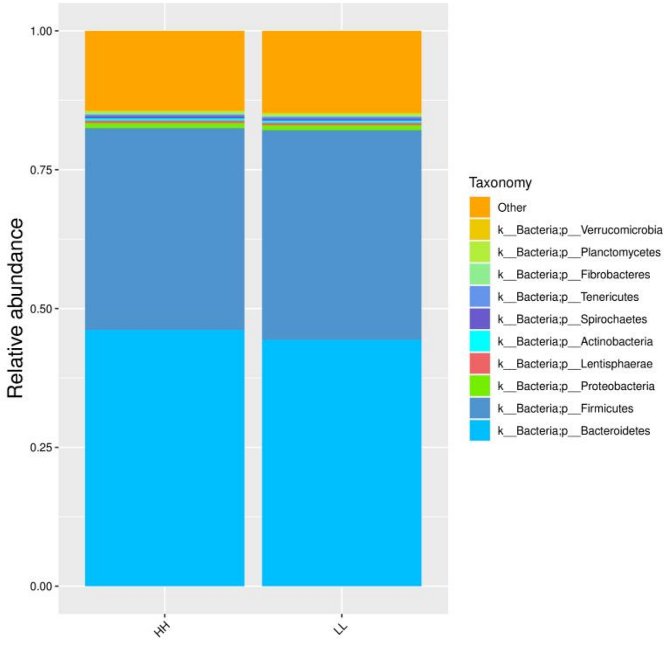

B. The main dominant bacterial genera

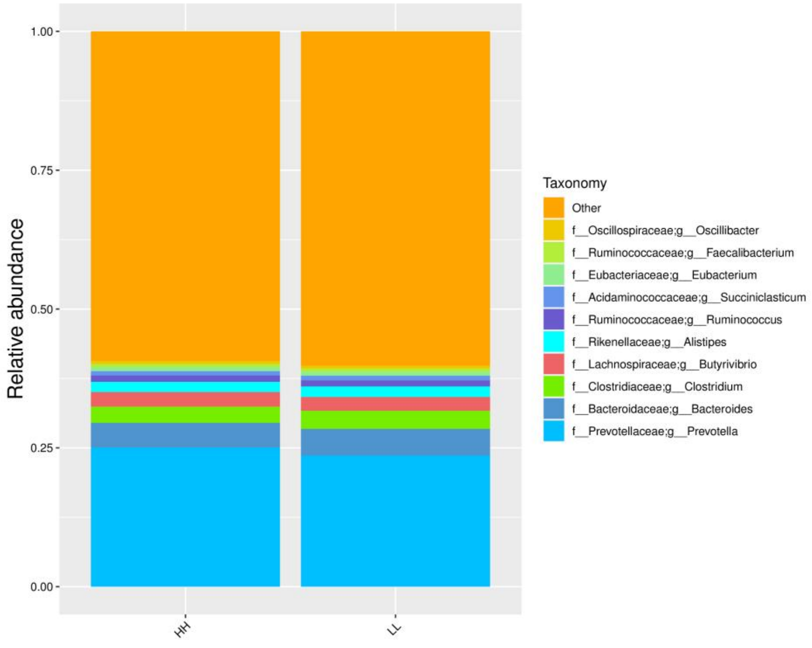

C. The main dominant bacterial species

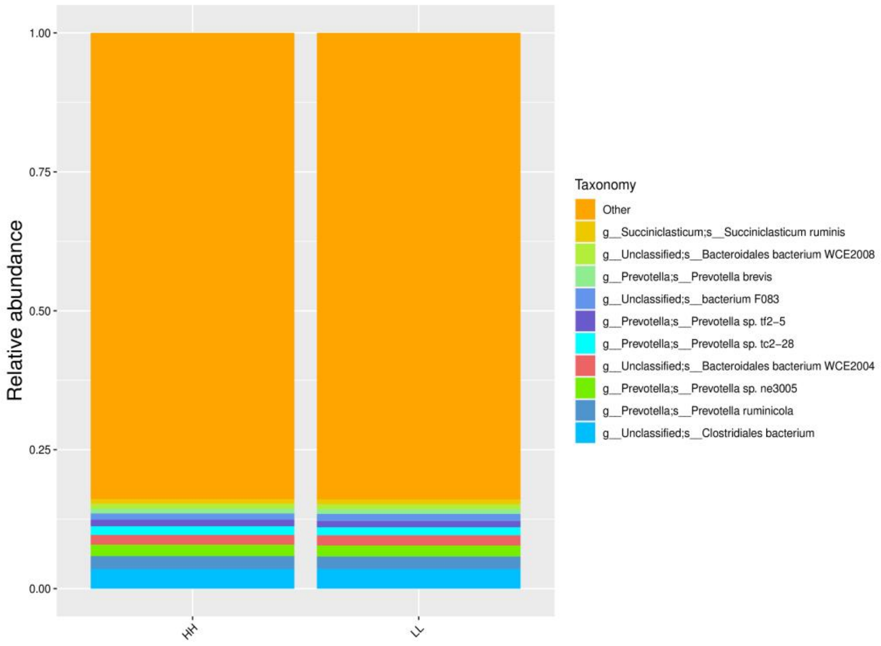

D. Significantly different bacterial phyla

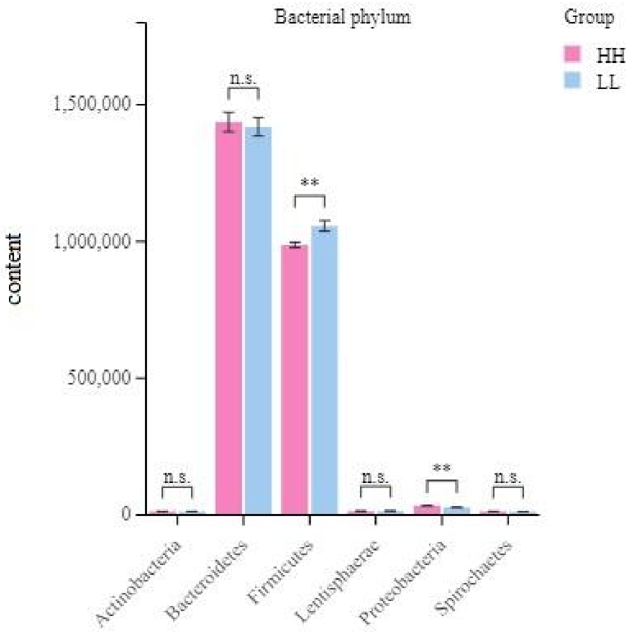

E. Significantly different bacterial genera

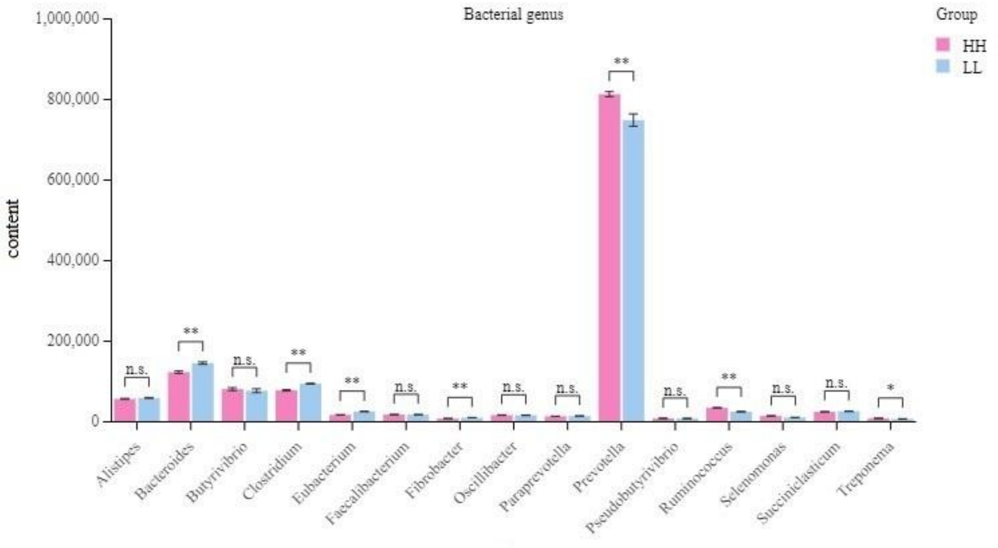

F. Significantly higher bacterial species in HH

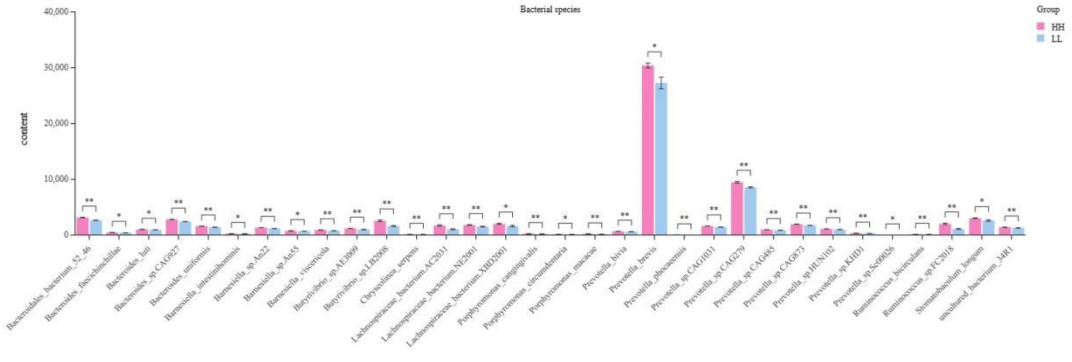

G. Significantly higher bacterial species in LL

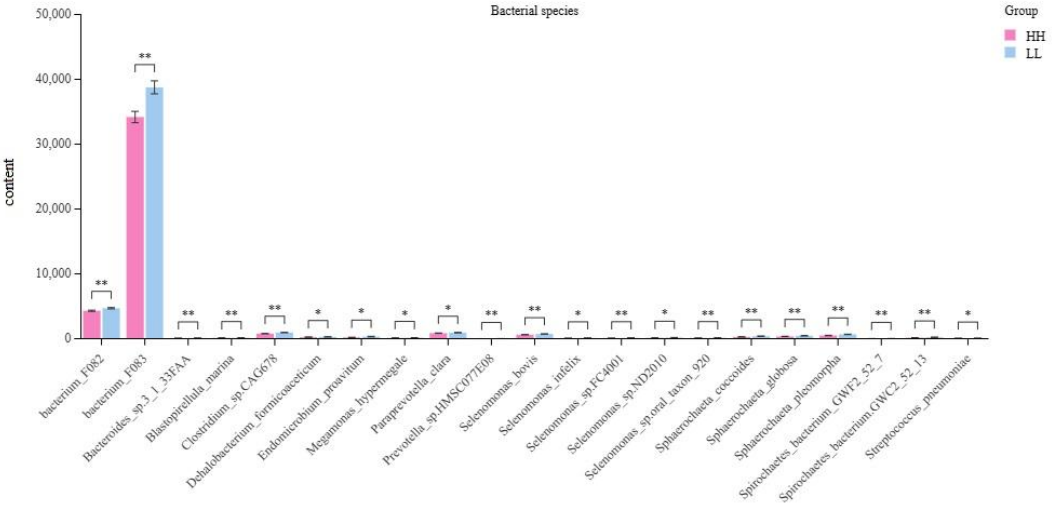

Supplement: Supplementary file 8 [file Image_2.pdf]
