# Supplementary material for: Multi-omics reveals the mechanism of rumen microbiome and its metabolome together with host metabolome participating in the regulation of milk production traits in dairy buffaloes
Source: Front Microbiol. 2024 Mar 8;15:1301292. doi: 10.3389/fmicb.2024.1301292 (PMC10959287; doi:10.3389/fmicb.2024.1301292)

Figure S3 Comparison of archaeal phyla, genera and species

A. The main dominant archaeal phyla

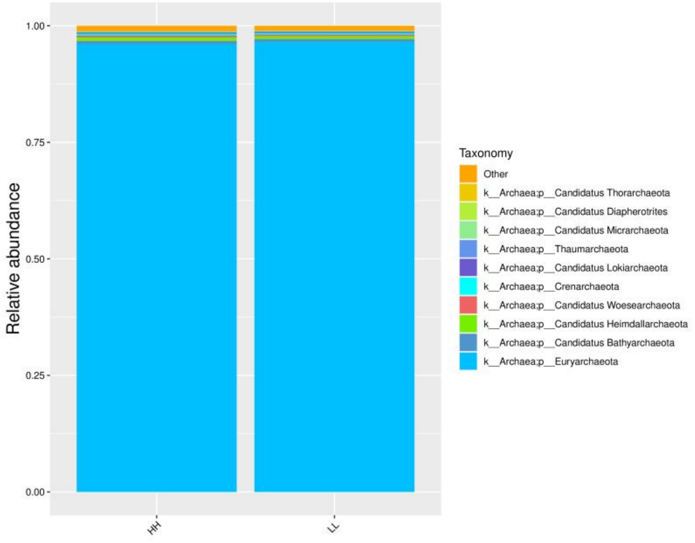

B. The main dominant archaeal genera

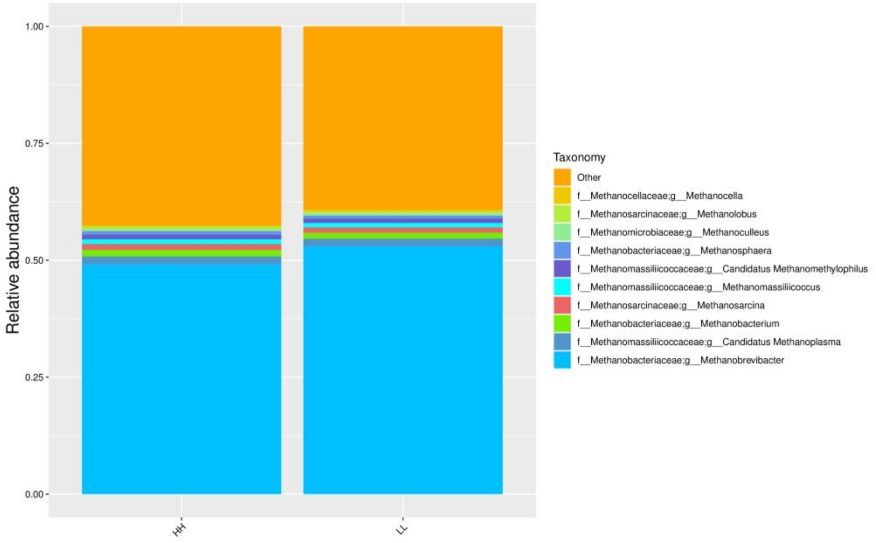

C. The main dominant archaeal species

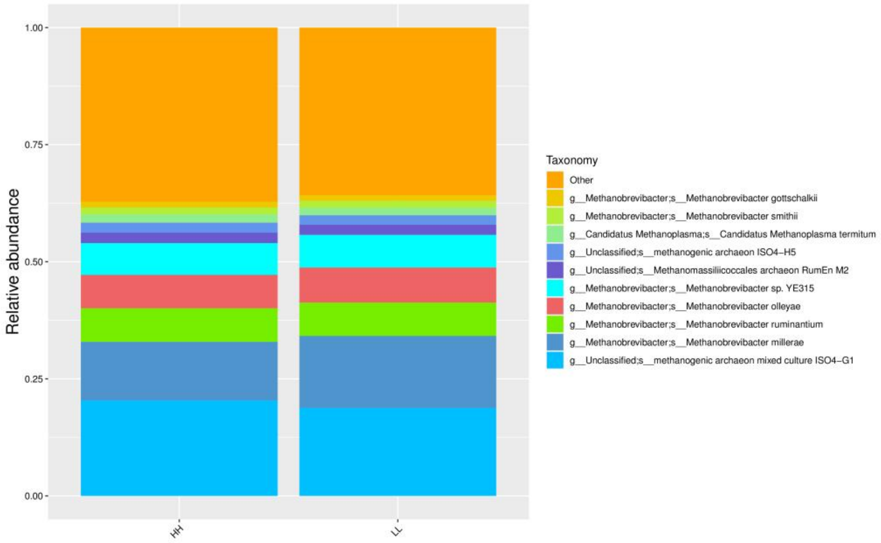

D. Significantly different archaeal phyla

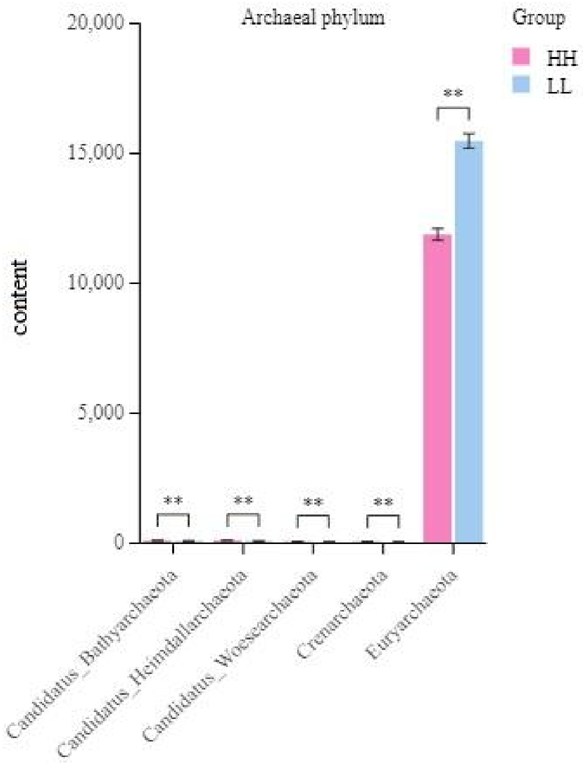

E. Significantly different archaeal genera

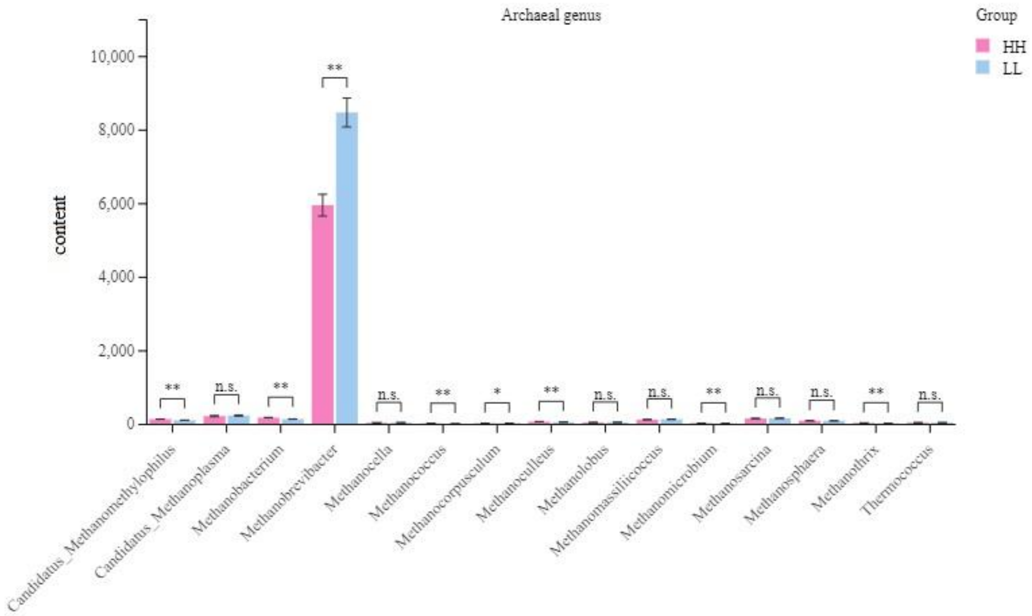

F. Significantly higher archaeal species in HH

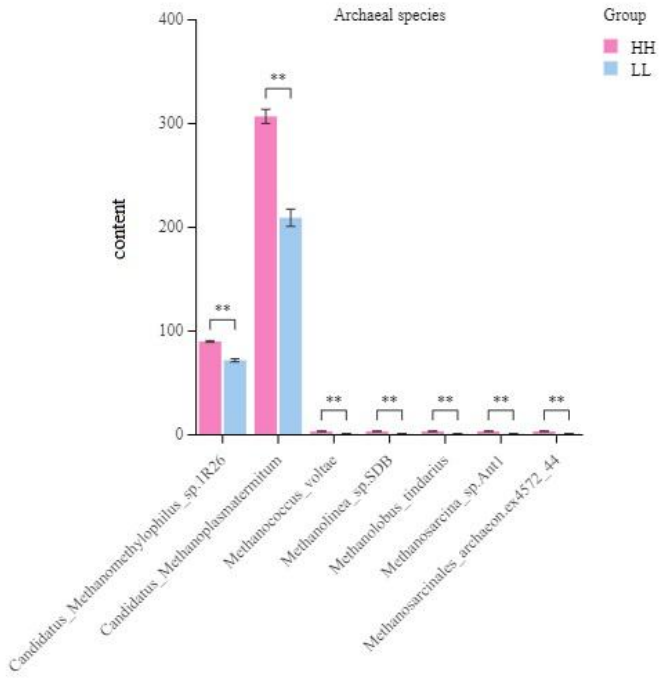

G. Significantly higher archaeal species in LL

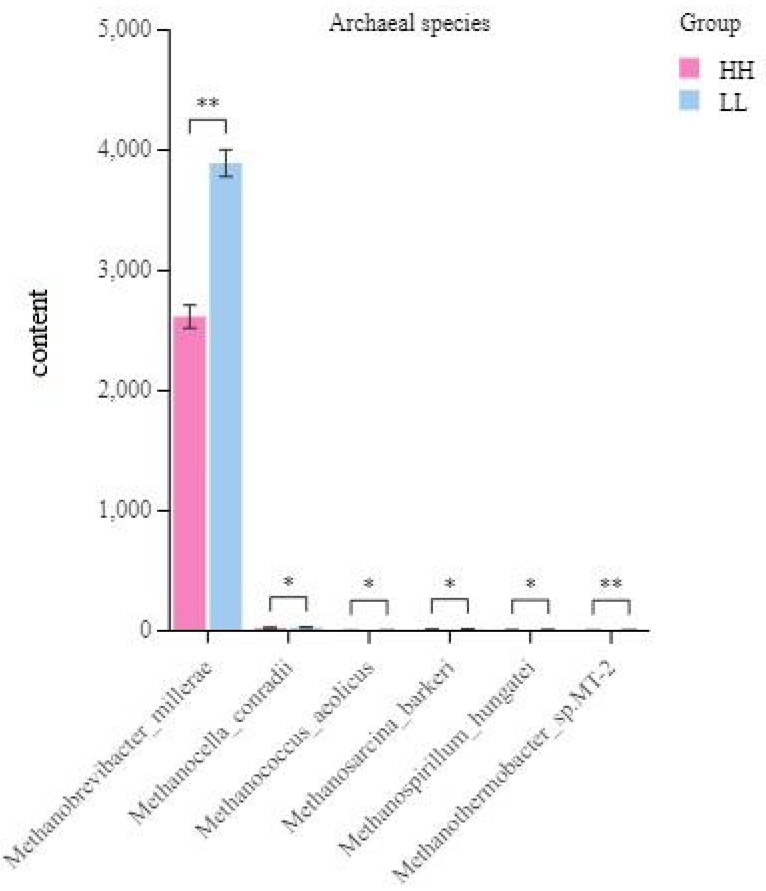

Supplement: Supplementary file 9 [file Image_3.pdf]
