# Supplementary material for: Multi-omics reveals the mechanism of rumen microbiome and its metabolome together with host metabolome participating in the regulation of milk production traits in dairy buffaloes
Source: Front Microbiol. 2024 Mar 8;15:1301292. doi: 10.3389/fmicb.2024.1301292 (PMC10959287; doi:10.3389/fmicb.2024.1301292)

Figure S5　Comparison of first-level and second-level KEGG pathways

A. The main dominant first-level

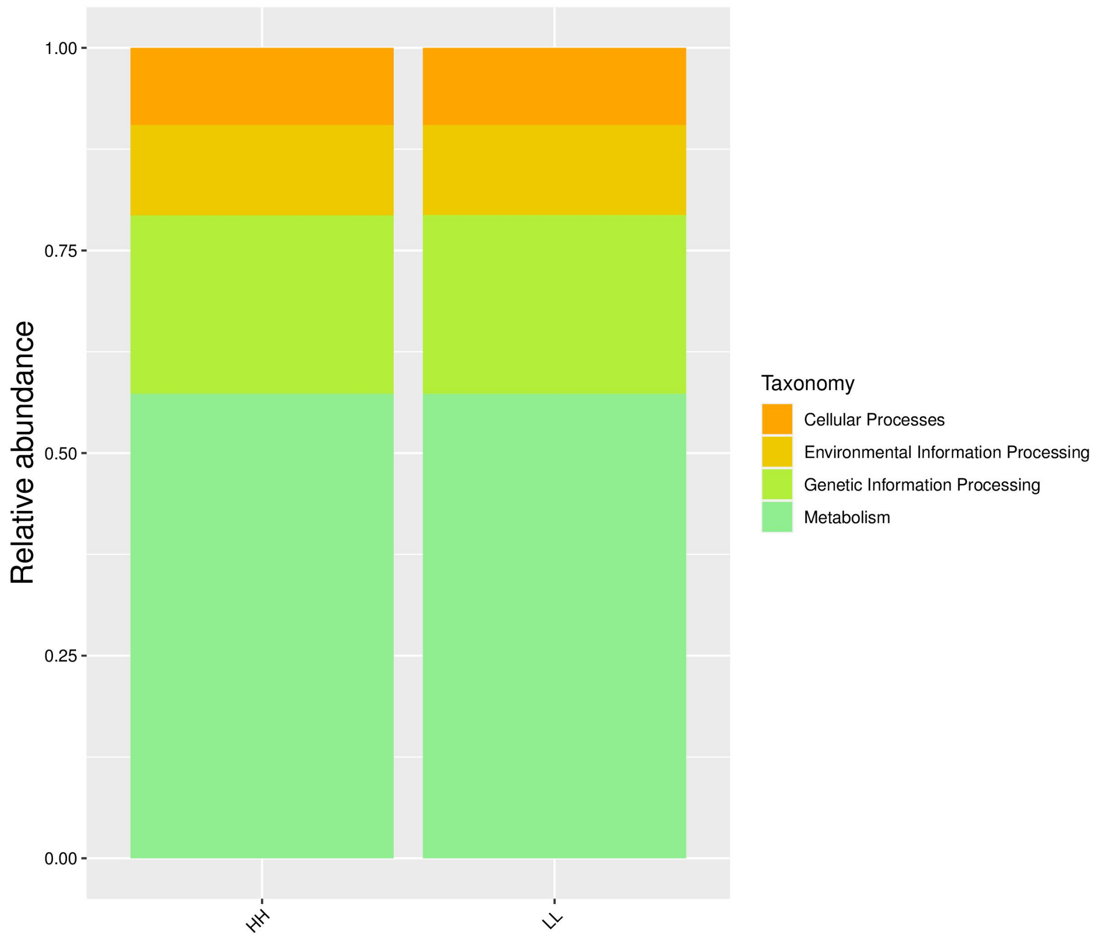

B. The main dominant second-level

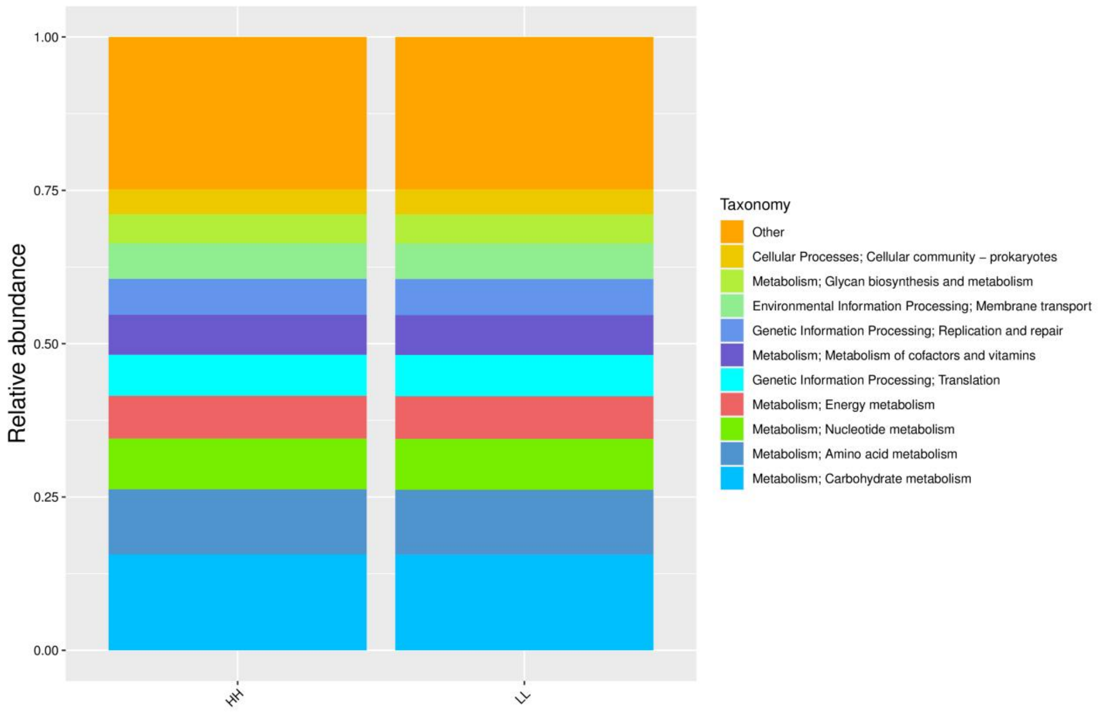

Supplement: Supplementary file 11 [file Image_5.pdf]
