# Supplementary material for: Multi-omics reveals the mechanism of rumen microbiome and its metabolome together with host metabolome participating in the regulation of milk production traits in dairy buffaloes
Source: Front Microbiol. 2024 Mar 8;15:1301292. doi: 10.3389/fmicb.2024.1301292 (PMC10959287; doi:10.3389/fmicb.2024.1301292)

Figure S6   Comparison of third-level pathways and functional modules

A. Third-level pathways significantly enriched in HH

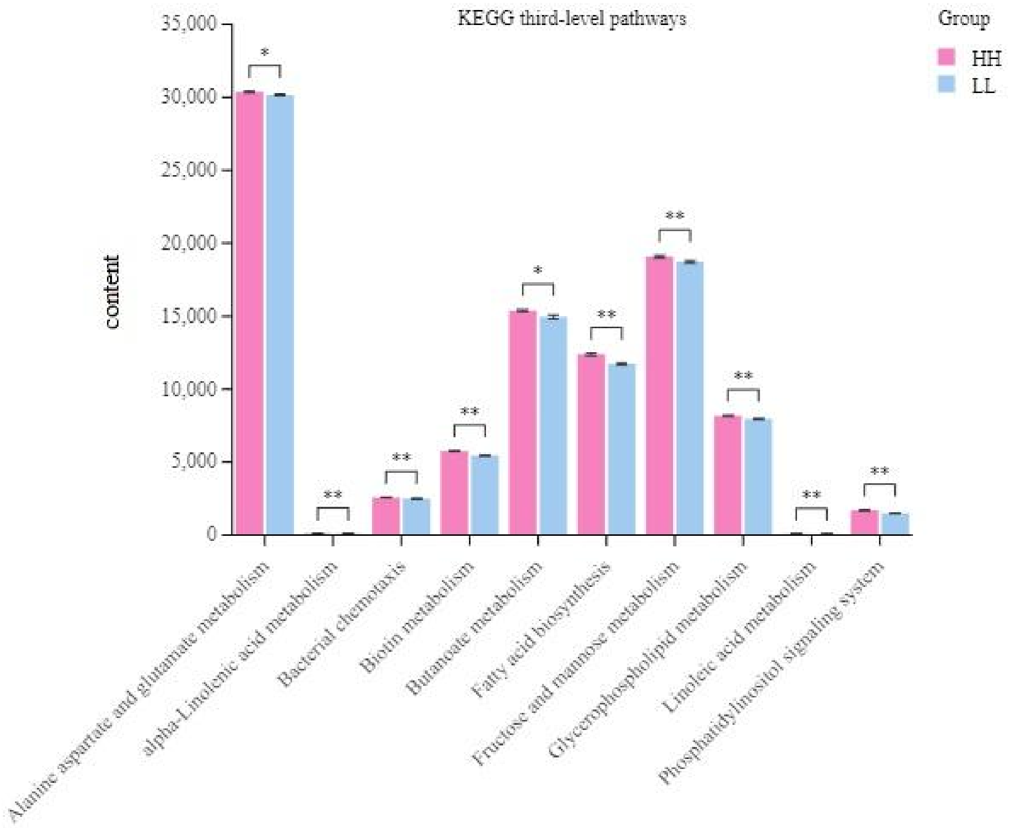

B. Functional modules significantly enriched in HH

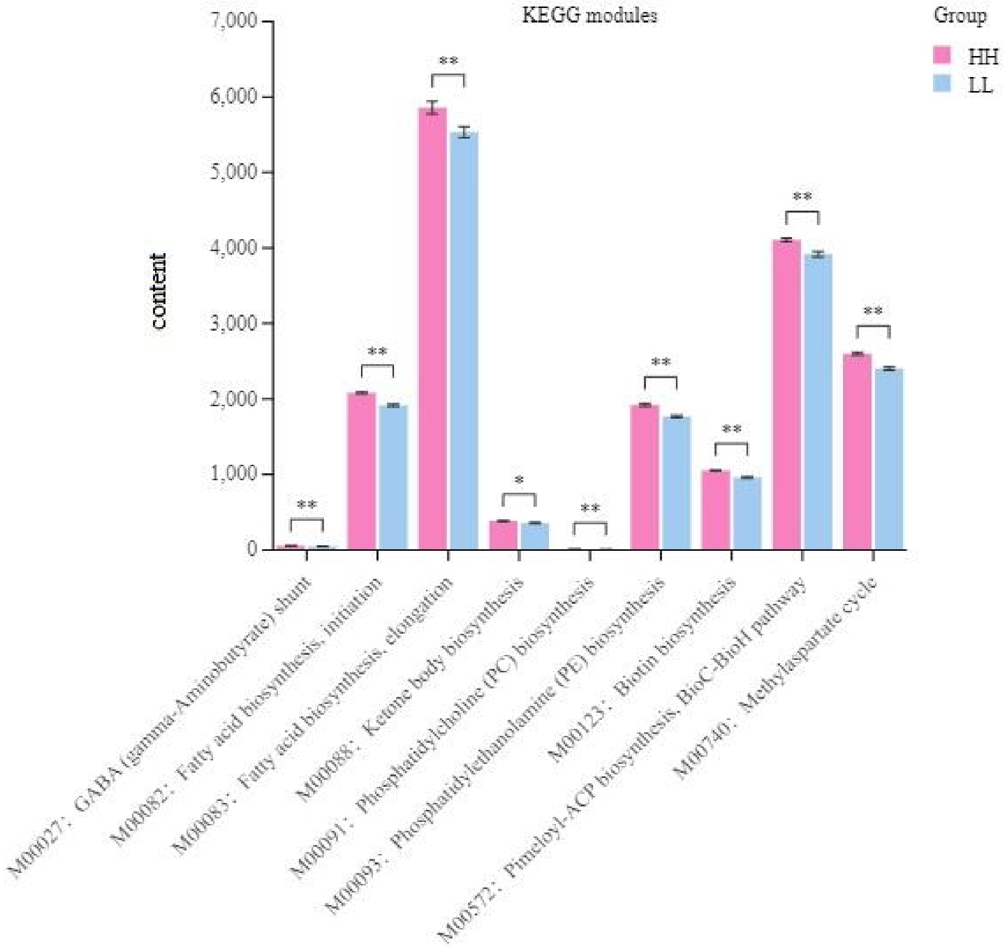

C. Third-level pathways significantly enriched in LL

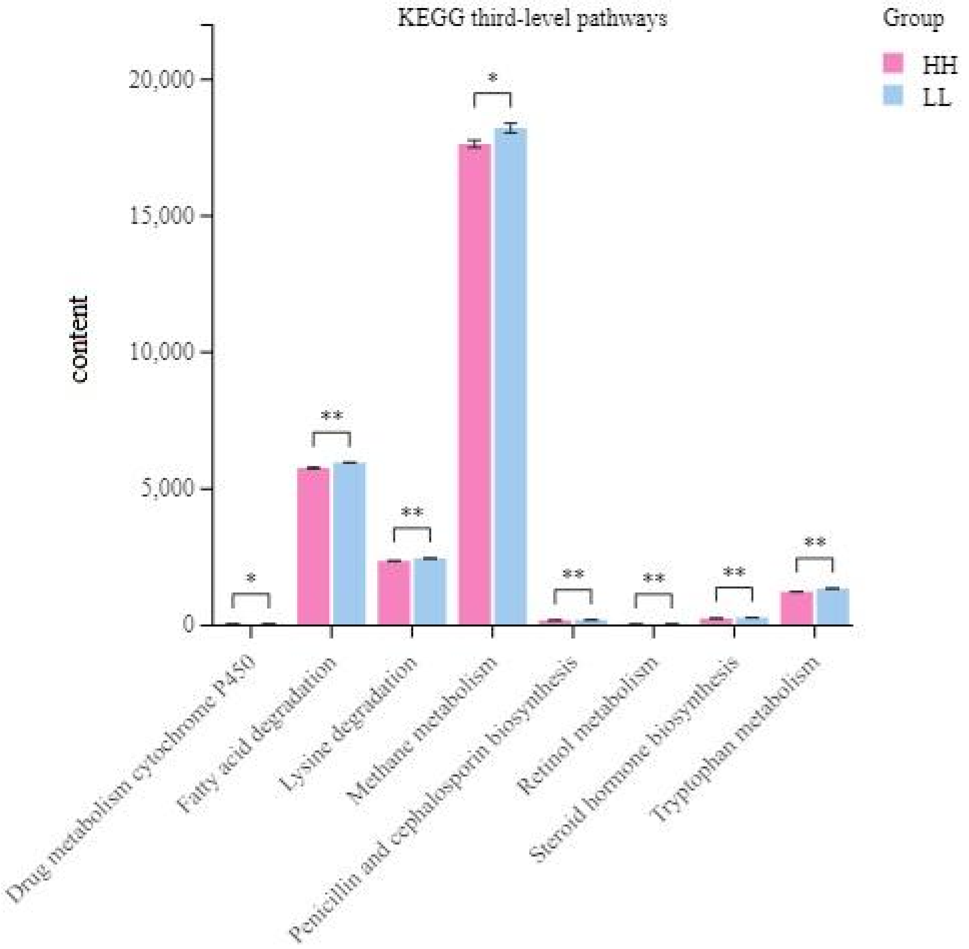

D. Functional modules significantly enriched in LL

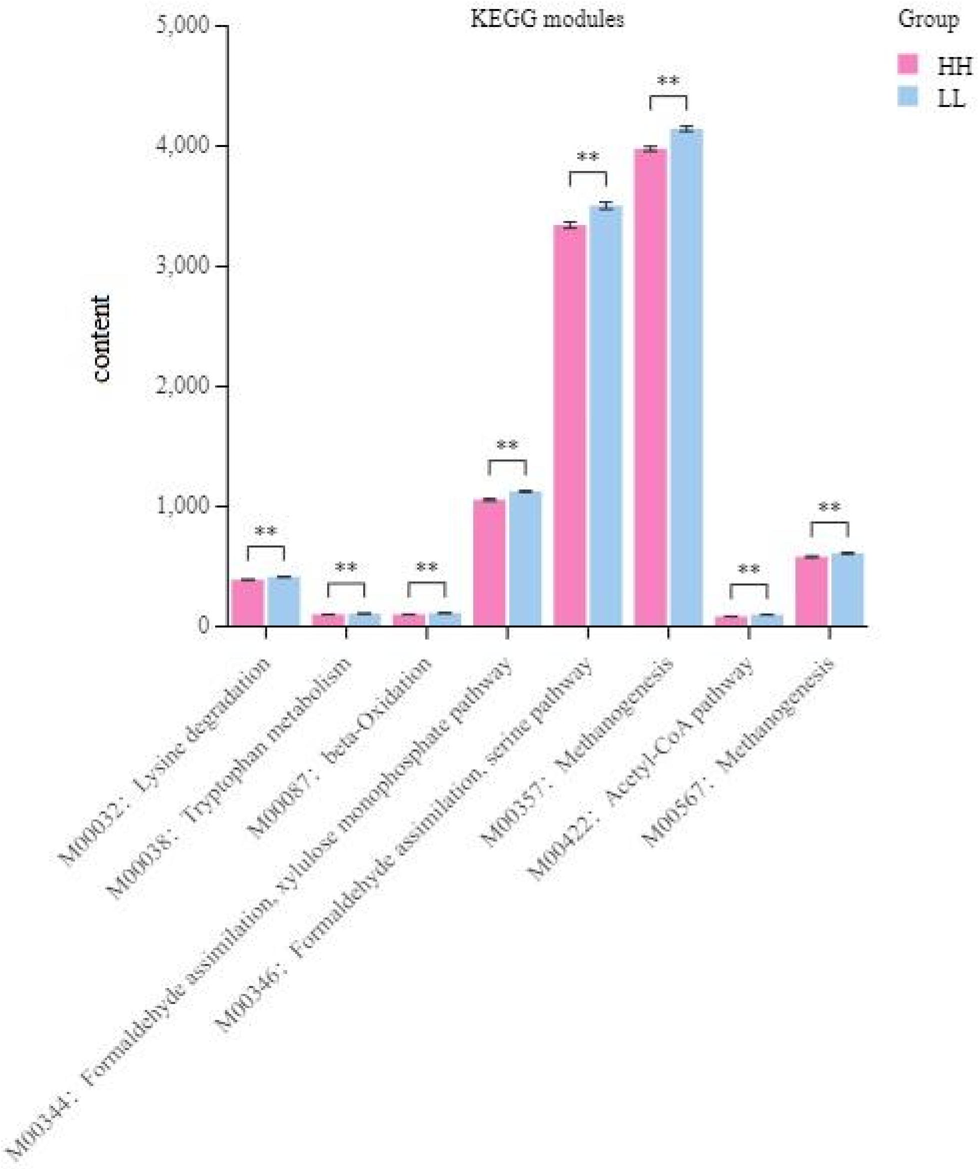

Supplement: Supplementary file 12 [file Image_6.pdf]
