# Supplementary material for: Multi-omics reveals the mechanism of rumen microbiome and its metabolome together with host metabolome participating in the regulation of milk production traits in dairy buffaloes
Source: Front Microbiol. 2024 Mar 8;15:1301292. doi: 10.3389/fmicb.2024.1301292 (PMC10959287; doi:10.3389/fmicb.2024.1301292)

Figure S8　Comparison of significantly enriched ECs involved in fatty acid biosynthesis and degradation

A. Fatty acid biosynthesis

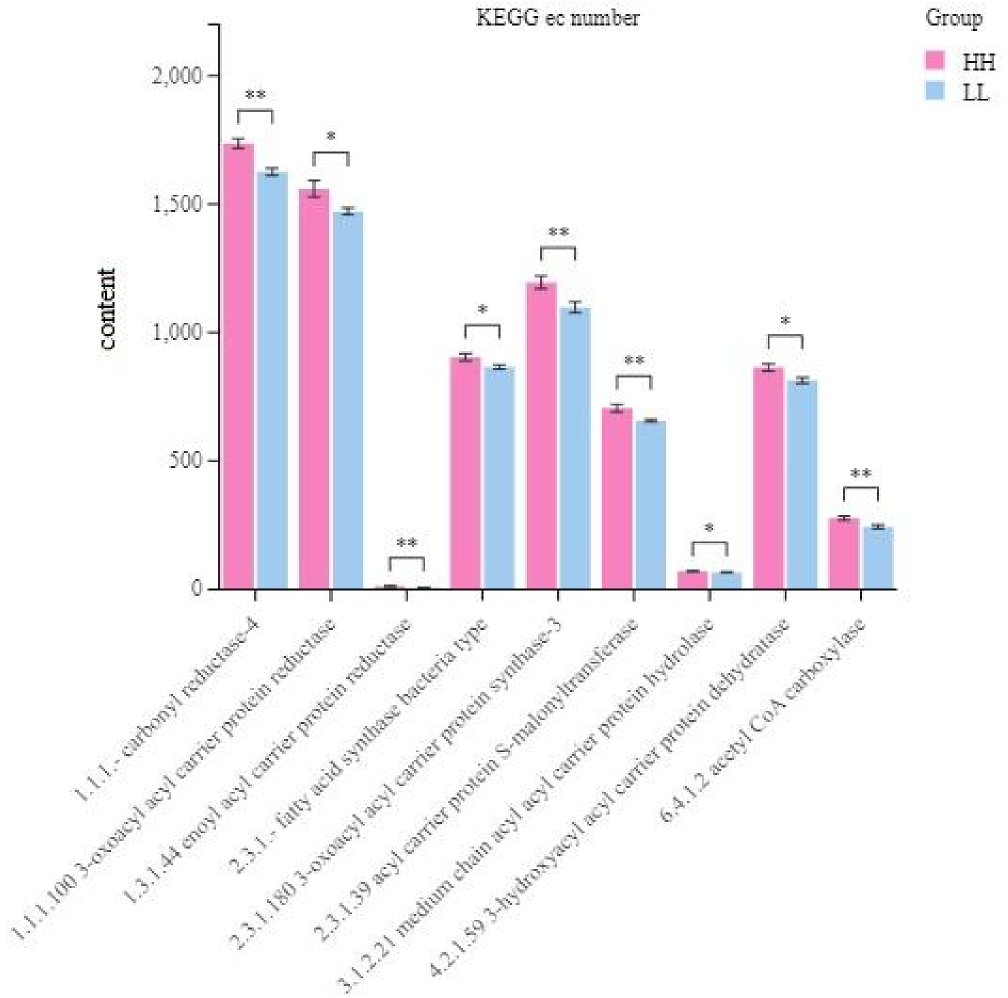

B. Fatty acid degradation

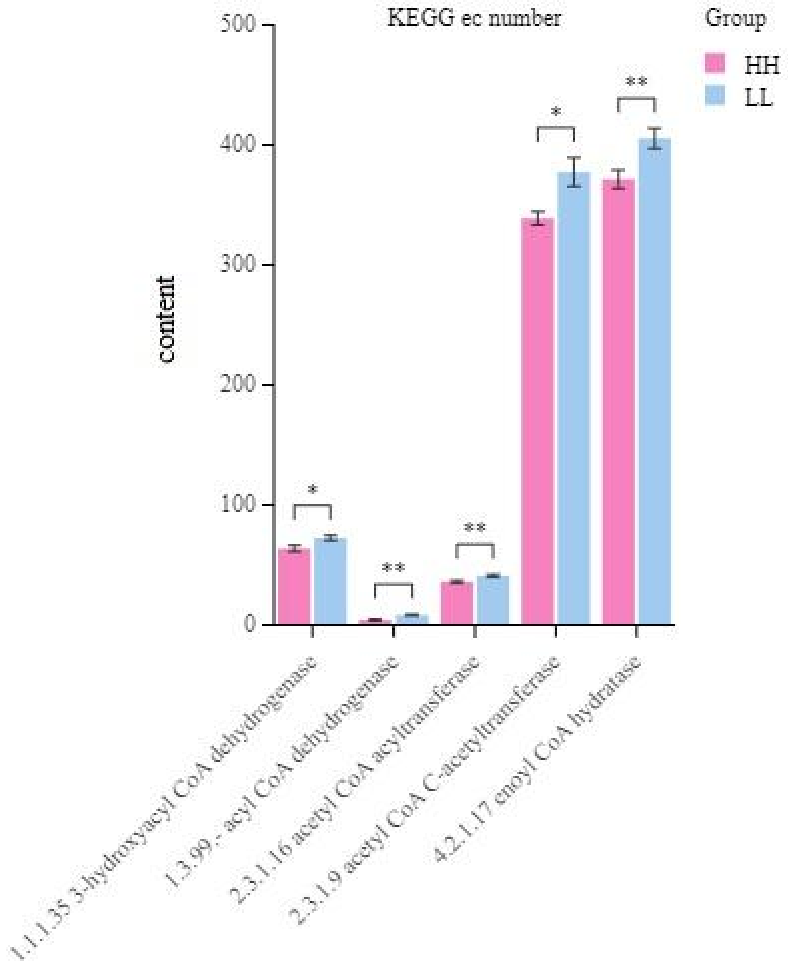

Supplement: Supplementary file 14 [file Image_8.pdf]
