# Supplementary figures and images for: Multi-omics reveals the mechanism of rumen microbiome and its metabolome together with host metabolome participating in the regulation of milk production traits in dairy buffaloes
Source: Front Microbiol. 2024 Mar 8;15:1301292. doi: 10.3389/fmicb.2024.1301292 (PMC10959287; doi:10.3389/fmicb.2024.1301292)

A. The Venn diagram shows comparison of metabolites between rumen and serum

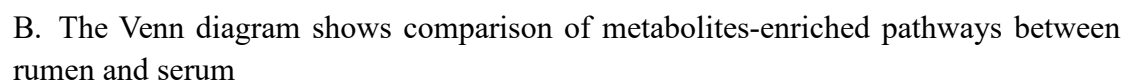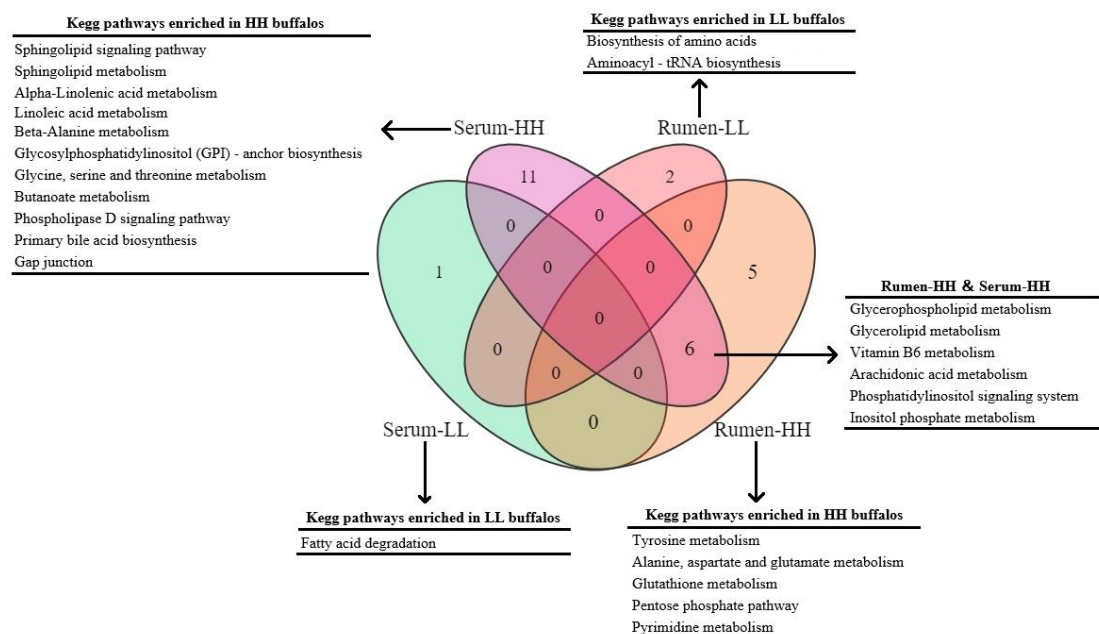

Supplement: Supplementary file 15 [file Image_9.pdf]
