# Supplementary material for: Multi-omics reveals the mechanism of rumen microbiome and its metabolome together with host metabolome participating in the regulation of milk production traits in dairy buffaloes
Source: Front Microbiol. 2024 Mar 8;15:1301292. doi: 10.3389/fmicb.2024.1301292 (PMC10959287; doi:10.3389/fmicb.2024.1301292)

Figure S10 Comparison of phenotype-associated metabolome between rumen and serum

The Venn diagram shows comparison of phenotype-associated metabolites-enriched pathways between rumen and serum

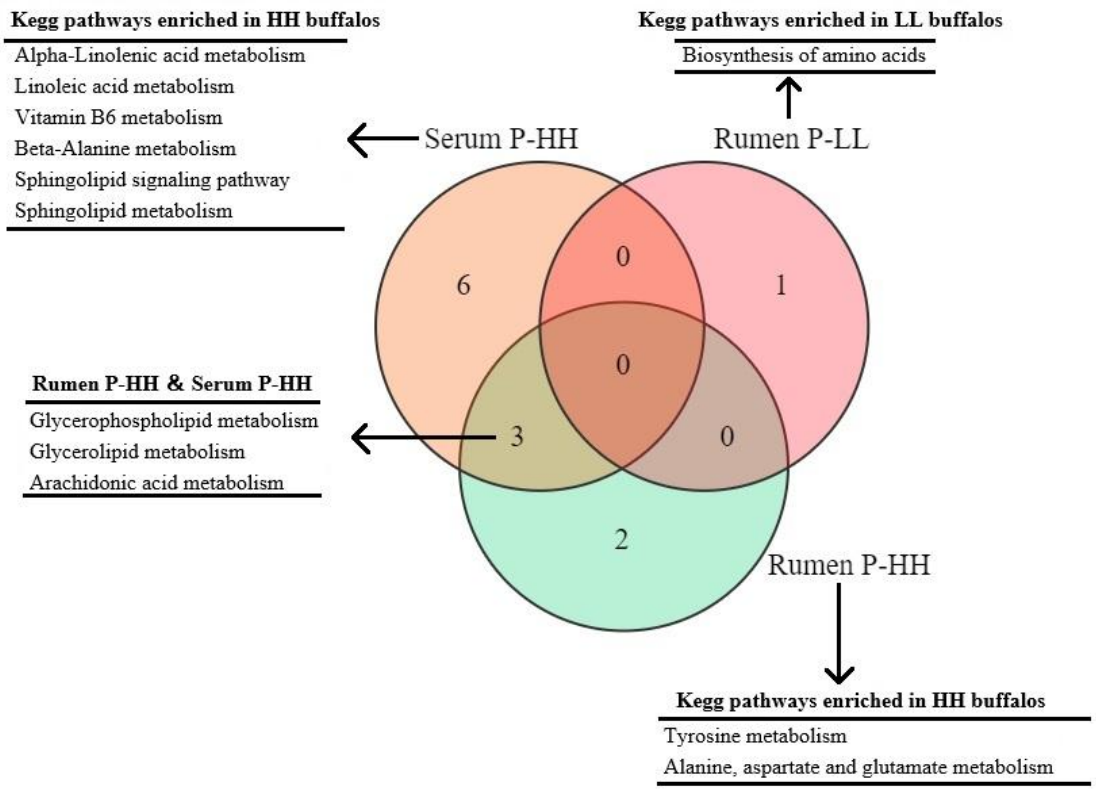

Supplement: Supplementary file 16 [file Image_10.pdf]
