# Supplementary material for: Multi-omics reveals the mechanism of rumen microbiome and its metabolome together with host metabolome participating in the regulation of milk production traits in dairy buffaloes
Source: Front Microbiol. 2024 Mar 8;15:1301292. doi: 10.3389/fmicb.2024.1301292 (PMC10959287; doi:10.3389/fmicb.2024.1301292)

Figure S11 Association heat map between phenotype-associated metabolites and microbiome functional modules

A. Spearman's correlations between microbiome functional modules and phenotype-associated metabolites in rumen

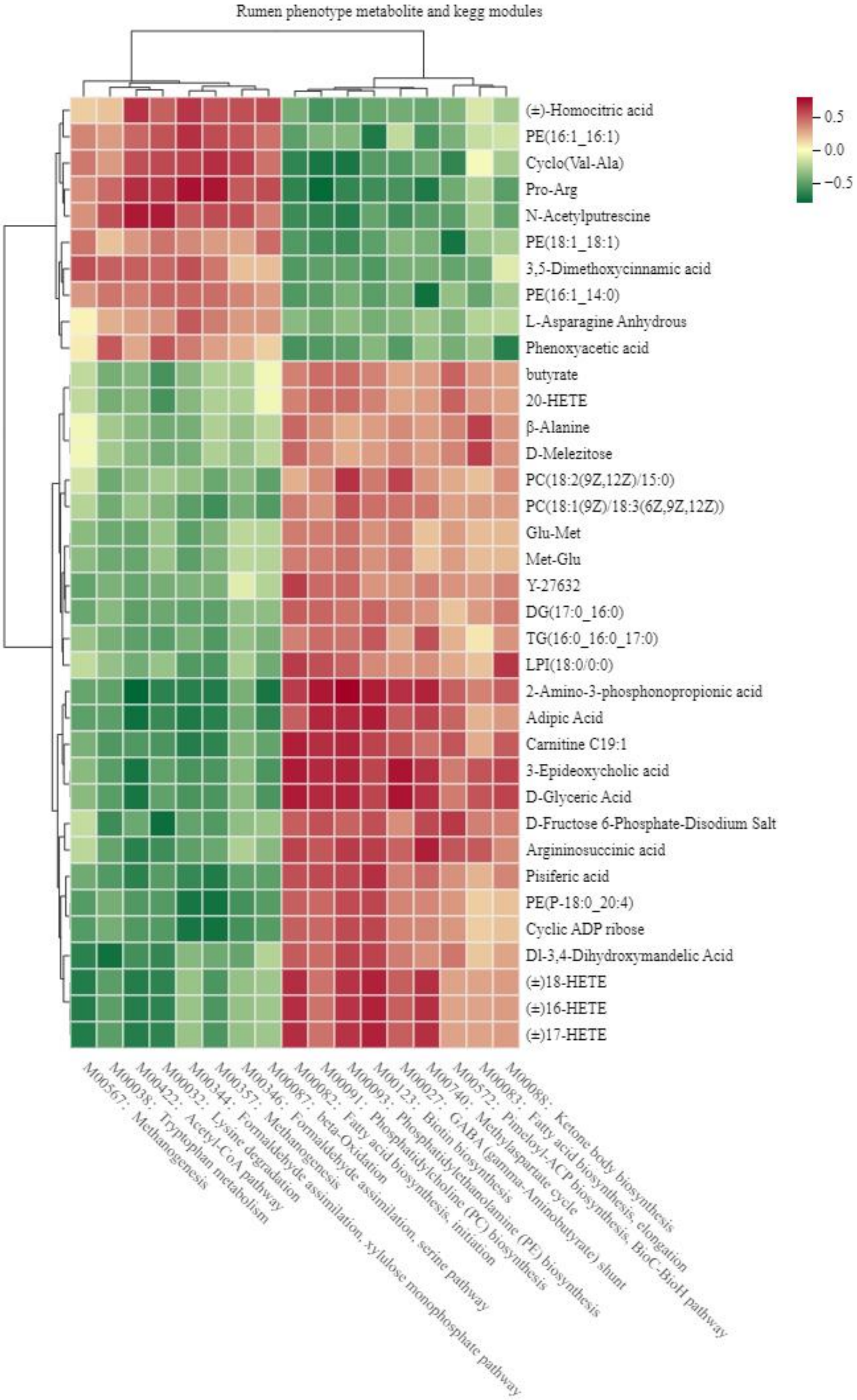

B. Spearman's correlations between microbiome functional modules and phenotype-associated metabolites in serum

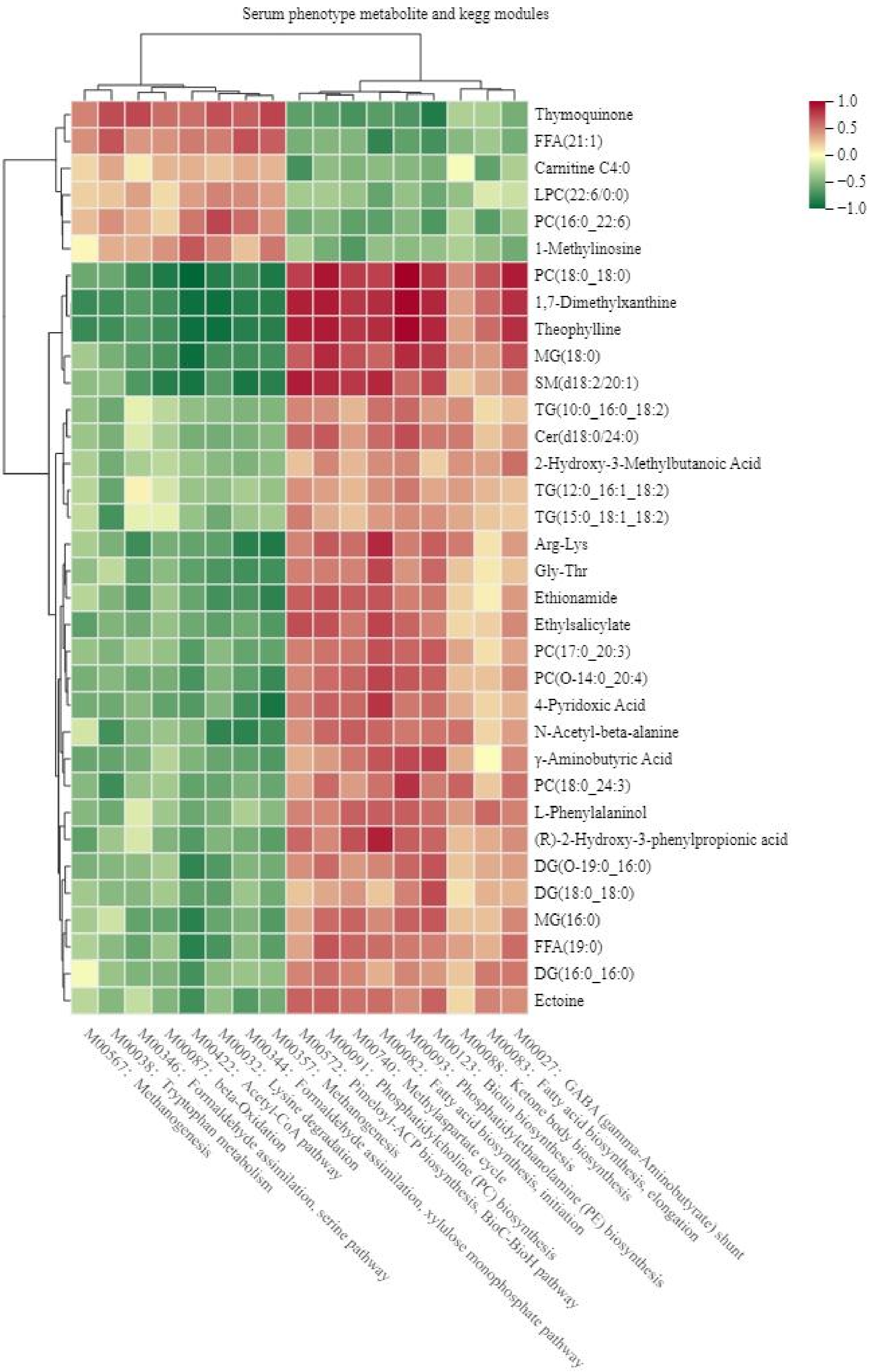

Supplement: Supplementary file 17 [file Image_11.pdf]
